# Human germline biallelic loss-of-function *OSMR* variants cause severe allergic disease

**DOI:** 10.70962/jhi.20260067

**Published:** 2026-05-28

**Authors:** Simran Samra, Mehul Sharma, Julia Körholz, Yihui Liu, Alyssa James, Christina Michalski, Pariya Yousefi, Kate L. Del Bel, Henry Y. Lu, Ashish A. Sharma, Maja Tarailo-Graovac, Joshua Dalmann, Lily Buder, Bhavi Modi, Ralf Wiedemuth, Liam Golding, Britt Drögemöller, Géraldine Blanchard-Rohner, Christof Senger, Wingfield Rehmus, Julie S. Prendiville, Massimo Mangino, Colin J. Ross, Clara D.M. van Karnebeek, Wyeth W. Wasserman, Sergio D. Rosenzweig, Julie Niemela, Pascal M. Lavoie, P. M. Prathibha, Oliver Wegehaupt, Catherine M. Biggs, Michael Boehnke, Leena Kinnunen, Heikki A. Koistinen, Margaret L. McKinnon, Jonas Maximillian Breuer, Jana Schönenkorb, Robert Brock, Sarah Thull, Christian Netzer, Clara Velmans, Norah Altuwaijri, Issam R. Hamadah, Ruqaiah Altassan, Ahmed Alfares, Sateesh Maddirevula, Siddaramappa Jagdish Patil, Diana K. Bayer, Jonathan J. Lyons, Stuart E. Turvey

**Affiliations:** 1 Department of Pediatrics, British Columbia Children’s Hospital, The University of British Columbia, Vancouver, Canada; 2 Experimental Medicine Program, Department of Medicine, The University of British Columbia, Vancouver, Canada; 3 Department of Pediatrics, Faculty of Medicine and University Hospital Carl Gustav Carus, Technische Universität Dresden, Dresden, Germany; 4 German Center for Child and Adolescent Health, Partner Site Leipzig/Dresden, Dresden, Germany; 5 Laboratory of Allergic Diseases, National Institute of Allergy and Infectious Diseases, National Institutes of Health, Bethesda, MD, USA; 6 Department of Pathology, Emory University, Atlanta, GA, USA; 7 Departments of Biochemistry, Molecular Biology and Medical Genetics, Alberta Children’s Hospital Research Institute, Cumming School of Medicine, University of Calgary, Calgary, Canada; 8 Department of Biochemistry and Medical Genetics, Children’s Hospital Research Institute of Manitoba, University of Manitoba, Winnipeg, Canada; 9 Unit of Immunology and Vaccinology, Division of General Pediatrics, Department of Pediatrics, Gynecology and Obstetrics, Geneva University Hospitals, University of Geneva, Geneva, Switzerland; 10 Department of Pathology and Laboratory Medicine, British Columbia Children’s Hospital, The University of British Columbia, Vancouver, Canada; 11 Department of Pediatrics and Dermatology, The University of British Columbia, Vancouver, Canada; 12 Department of Pharmaceutical Sciences, University of British Columbia, Vancouver, Canada; 13 Emma Center for Personalized Medicine, Department of Pediatrics and Human Genetics, Amsterdam University Medical Center, Amsterdam Gastroenterology and Metabolism, University of Amsterdam, Amsterdam, Netherlands; 14 Centre for Molecular Medicine and Therapeutics, British Columbia Children’s Hospital Research Institute, Vancouver, Canada; 15 Department of Medical Genetics, The University of British Columbia, Vancouver, Canada; 16 Immunology Service, Clinical Center, National Institutes of Health, Bethesda, MD, USA; 17 Department of Dermatology, Narayana Hrudayalaya Hospital, Mazumdar-Shaw Medical Center, Bangalore, India; 18 Center for Chronic Immunodeficiency, Medical Center, Faculty of Medicine, Institute for Immunodeficiency, University of Freiburg, Freiburg, Germany; 19 Division of Pediatric Hematology and Oncology, Department of Pediatrics and Adolescent Medicine, Medical Center, Faculty of Medicine, University of Freiburg, Freiburg, Germany; 20 Department of Biostatistics, Center of Statistical Genetics, University of Michigan, Ann Arbor, MI, USA; 21 Department of Public Health and Welfare, Finnish Institute for Health and Welfare, Helsinki, Finland; 22 Research Programs Unit, Faculty of Medicine, Clinical and Molecular Metabolism, University of Helsinki, Helsinki, Finland; 23 Department of Medicine, University of Helsinki and Helsinki University Hospital, Helsinki, Finland; 24 Minerva Foundation Institute for Medical Research, Helsinki, Finland; 25 Pediatric Pulmonology and Allergology, Faculty of Medicine and University Hospital Cologne, University Children’s Hospital Cologne, University of Cologne, Cologne, Germany; 26 Centre for Rare Diseases, Faculty of Medicine and University Hospital Cologne, University of Cologne, Germany; 27 Faculty of Medicine and University Hospital Cologne, Institute of Human Genetics, University of Cologne, Cologne, Germany; 28 Department of Dermatology, King Faisal Specialist Hospital and Research Center, Riyadh, Saudi Arabia; 29 Medical Genomic Department, Genomic Medicine Center of Excellence, King Faisal Specialist Hospital and Research Center, Riyadh, Saudi Arabia; 30 Genomic Medicine Center of Excellence, King Faisal Specialist Hospital & Research Centre, Riyadh, Saudi Arabia; 31 King Faisal Specialist Hospital and Research Center, Medina, Saudi Arabia; 32 Division of Medical Genetics, Narayana Hrudayalaya Hospital, Mazumdar-Shaw Medical Center, Bangalore, India; 33 Department of Pediatrics, University of Iowa Stead Family Children’s Hospital, Iowa City, IA, USA; 34 Division of Allergy & Immunology, Department of Medicine, University of California San Diego, La Jolla, CA, USA; 35 Veterans Affairs San Diego Healthcare System, La Jolla, CA, USA

## Abstract

Oncostatin M (OSM) receptor beta (OSMRβ), encoded by *OSMR*, is a cytokine receptor subunit required for signaling by OSM and IL-31. We identified 10 affected individuals from seven unrelated families with germline biallelic loss-of-function variants in *OSMR* who shared a phenotype of early-onset, severe, widespread atopic dermatitis with peripheral eosinophilia and markedly elevated serum IgE. All patient-derived OSMRβ variants failed to localize to the cell surface, resulting in selective loss of OSM-dependent signaling. Patient cells showed markedly reduced OSM-induced phosphorylation of STAT1, STAT3, and STAT5, while signaling through other IL-6 family receptor complexes remained intact. Transcriptomic profiling of patient primary dermal fibroblasts revealed consistent downstream effects, including loss of interferon-responsive and inflammatory gene programs. Re-expression of wild-type *OSMR* restored receptor surface expression, STAT activation, and transcriptional responses, confirming a causal loss-of-function mechanism. Together, these findings establish biallelic *OSMR* deficiency as a novel primary atopic disorder.

## Teaser

Samra, Sharma, et al. identify biallelic loss-of-function variants in *OSMR* as the cause of a new human primary atopic disorder characterized by severe early-onset eczema, eosinophilia, and elevated IgE. Loss of OSMRβ surface expression abolishes OSM-dependent STAT signaling, revealing a key role for OSM-OSMR signaling in maintaining cutaneous immune homeostasis.

## Graphical Abstract

**Figure:**
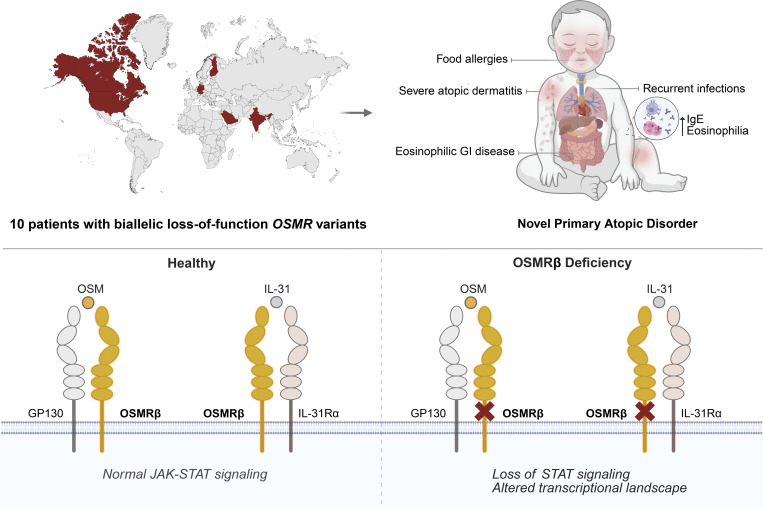

## Introduction

Inborn errors of immunity (IEIs) are a group of monogenic disorders that impair or dysregulate the function of the human immune system (1, 2). Primary atopic disorders (PADs) are a subset of the IEIs where severe allergic disease is the predominant clinical feature (3). The discovery of new PADs advances our understanding of the molecular mechanisms responsible for allergic inflammation and can transform the clinical care of affected individuals (3, 4, 5).

In this study, we describe a novel human PAD caused by germline biallelic loss-of-function (LOF) variants in the gene *OSMR* found in 10 patients from seven unrelated families spanning three continents. Oncostatin M (OSM) receptor beta (OSMRβ), encoded by *OSMR* (Online Mendelian Inheritance in Man [OMIM]: 601743), is a member of the interleukin (IL)-6 superfamily. OSMRβ functions as a shared receptor component in two cytokine signaling pathways, pairing with GP130 to form the OSM type II receptor and with IL-31Rα to form the IL-31 receptor, thereby mediating the biological effects of both OSM (OMIM: 165095) and IL-31 (OMIM: 609509). OSM is a monomeric glycoprotein produced by activated T cells, monocytes (6), macrophages (7), and neutrophils (8, 9), whereas IL-31 is mainly produced by CD4^+^ T helper cells (10). Human biallelic OSM deficiency causes a bone marrow failure syndrome (11, 12). The expression of the IL-31 receptor is specifically high in the dorsal root ganglion and is recognized for promoting itch in prurigo nodularis (13) and other pruritic skin conditions, while OSMRβ is widely expressed in a variety of cell types and tissues, including epithelial cells, fibroblasts, blood vessels, nerve cells, respiratory tissues, adipose tissues, and lymph nodes (14, 15). OSMRβ also plays a role in keratinocyte proliferation, differentiation, and inflammatory responsiveness (16). Consistent with its broad tissue expression, dysregulated OSMRβ signaling has been implicated in diverse inflammatory, fibrotic, and epithelial diseases (17), including inflammatory bowel disease (18, 19, 20), pulmonary fibrosis (21), inflammatory skin conditions such as IL-31-mediated pruritus (22), diffuse cutaneous systemic sclerosis (23), cutaneous T cell lymphoma (24), chronic autoimmune urticaria (25), and familial primary localized cutaneous amyloidosis (FPLCA) (26, 27, 28, 29, 30, 31). FPLCA is an extremely pruritic skin disorder characterized by amyloid deposits in the superficial dermis. Heterozygous variants in *IL31RA* and *OSMR* are associated with FPLCA, which is typically an autosomal dominant disorder with onset of symptoms in adolescence or adulthood (26, 27, 28, 29, 30, 31). Notably, pathogenic *OSMR* variants have previously been described exclusively in the context of autosomal dominant FPLCA, although a single patient with recessive OSMR deficiency presenting with systemic allergic disease was reported while this manuscript was under peer review (32).

Here we expand our understanding of the role of OSMRβ in human health and disease by describing a cohort of 10 patients with biallelic LOF variants in *OSMR* causing a new PAD.

## Results

### Identification of patients with biallelic *OSMR* deficiency and severe early-onset atopic disease

We investigated 10 patients from seven kindreds with severe early-onset atopic disease that is most notable for treatment-resistant atopic dermatitis. Patients were identified by their clinicians as candidates for assessment of a monogenic disorder based on their severe phenotype (as detailed in the next section). The patients were of self-reported European (kindreds A, B, and C), South Asian (kindreds D and G), or Arab ancestry (kindreds E and F). All patients carried biallelic variants in *OSMR* (NM_003999.3). By sequencing their healthy parents and siblings (when available), we established an autosomal recessive pattern of inheritance.

**Figure 1.**
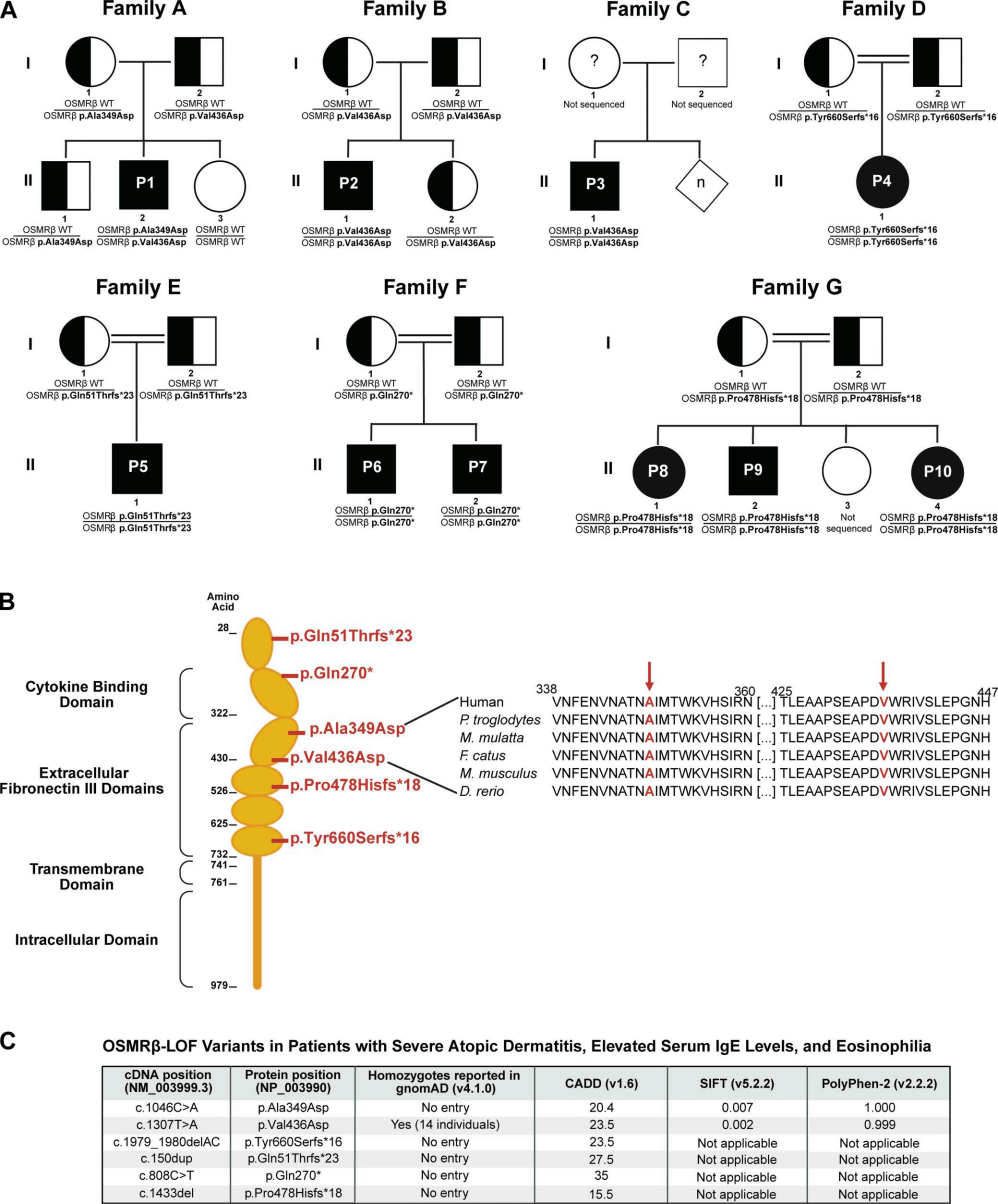
**10** **patients with severe allergic disease and *OSMR* variants. (A)** Family pedigrees of the 10 patients from seven different families. Filled symbols = affected individuals; unfilled symbols = unaffected individuals; half-filled symbols = heterozygous unaffected individuals; diamond symbol = unspecified sex. **(B)** Schematic illustrating the protein domains of OSMRβ. The amino acid locations of the two missense and four premature stop variants are shown in red. The region surrounding the missense variants (p.Ala349Asp and p.Val436Asp) was aligned with sequences from other species and found to be evolutionarily conserved. **(C)** LOF *OSMR* variants reported in patients. To our knowledge, only the p.Val436Asp variant is reported for homozygous carriers in gnomAD (https://gnomad.broadinstitute.org/), a large population database. The following in silico tools predicted the *OSMR* LOF variants to be damaging: Combined Annotation Dependent Depletion (CADD) (https://cadd.gs.washington.edu/), Sorting Intolerant from Tolerant (SIFT) (https://siftdna.org/), and Polymorphism Phenotyping v2 (PolyPhen-2) (https://bio.tools/polyphen-2). SIFT and PolyPhen-2 cannot be applied to premature stop and frameshift variants.

Patient 1 (P1) (from kindred A) carried compound heterozygous missense *OSMR* variants (c.1046C>A, p.Ala349Asp and c.1307T>A, p.Val436Asp). The p.Ala349Asp variant was maternally inherited, while the p.Val436Asp variant was paternally inherited. Patients 2 and 3 (P2 and P3) (from kindreds B and C, respectively) were homozygous for the c.1307T>A, p.Val436Asp *OSMR* variant. P2 inherited the variants from their healthy heterozygous parents, while no pedigree information was available for P3. Patient 4 (P4) (from kindred D) was homozygous for the c.1979_1980delAC, p.Tyr660Serfs*16 *OSMR* variant. Patient 5 (P5) (from kindred E) was homozygous for the c.150dup, p.Gln51Thrfs*23 *OSMR* variant. Patients 6 and 7 (P6 and P7) (from kindred F) are siblings and were homozygous for the c.808C>T, p.Gln270* *OSMR* variant. Patients 8–10 (P8, P9, and P10) (from kindred G) are siblings and were homozygous for the c.1433del, p.Pro478Hisfs*18 *OSMR* variant. Patients 4–10 inherited their biallelic *OSMR* variants from their healthy heterozygous consanguineous parents (Fig. 1 A).

All identified variants localized to either the cytokine-binding domain or the extracellular fibronectin III domains of OSMRβ, regions required for receptor signaling. The fibronectin III domains, in particular, are essential for receptor dimerization and downstream signal transduction (33, 34) (Fig. 1 B). Both the p.Ala349Asp and p.Val436Asp variants result in the substitution of a nonpolar amino acid (alanine and valine, respectively) with a negatively charged aspartic acid at evolutionarily conserved positions. Both variants were predicted to be damaging by a variety of in silico tools (Fig. 1, B and C). The remaining *OSMR* variants, p.Gln51Thrfs*23, p.Gln270*, p.Pro478Hisfs*18, and p.Tyr660Serfs*16, are predicted LOF variants that introduce premature termination codons (Fig. 1 C).

### Unifying clinical features of the 10 patients with biallelic *OSMR* deficiency

**Figure 2.**
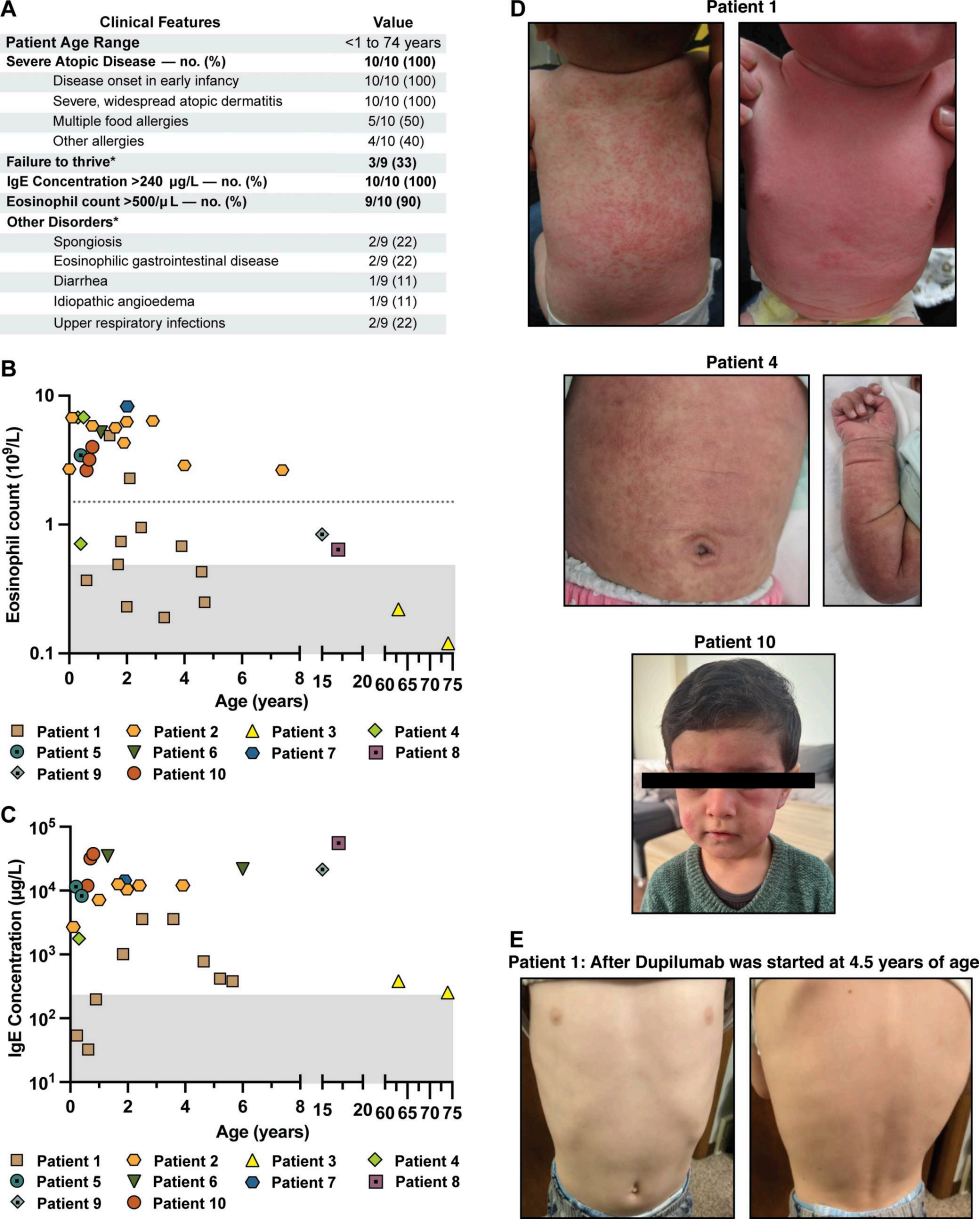
**Major clinical features of the 10 patients. (A)** Tabulation and comparison of the clinical phenotype for 10 patients. *Please note that for P3, we had limited information about his family pedigree or childhood growth trajectory. **(B)** Eosinophil count in whole blood. The shaded area represents counts <0.5 × 10^9^/liter, which is the typical upper limit of normal. The horizontal broken line denotes an eosinophil count of 1.5 × 10^9^/liter, since hypereosinophilic syndrome is traditionally defined as sustained peripheral blood eosinophilia >1.5 × 10^9^/liter. **(C)** IgE concentration in whole blood. The shaded area represents IgE <240 μg/l, which is the typical upper limit of normal. **(D)** Photographs of widespread and severe atopic dermatitis in P1 and P4 during infancy. Photograph of P10’s facial skin, which shows extensive acute dermatitis with periorbital swelling. **(E)** Photographs of P1’s skin, which dramatically improved after dupilumab was started at 4.5 years of age.

**Figure S1.**
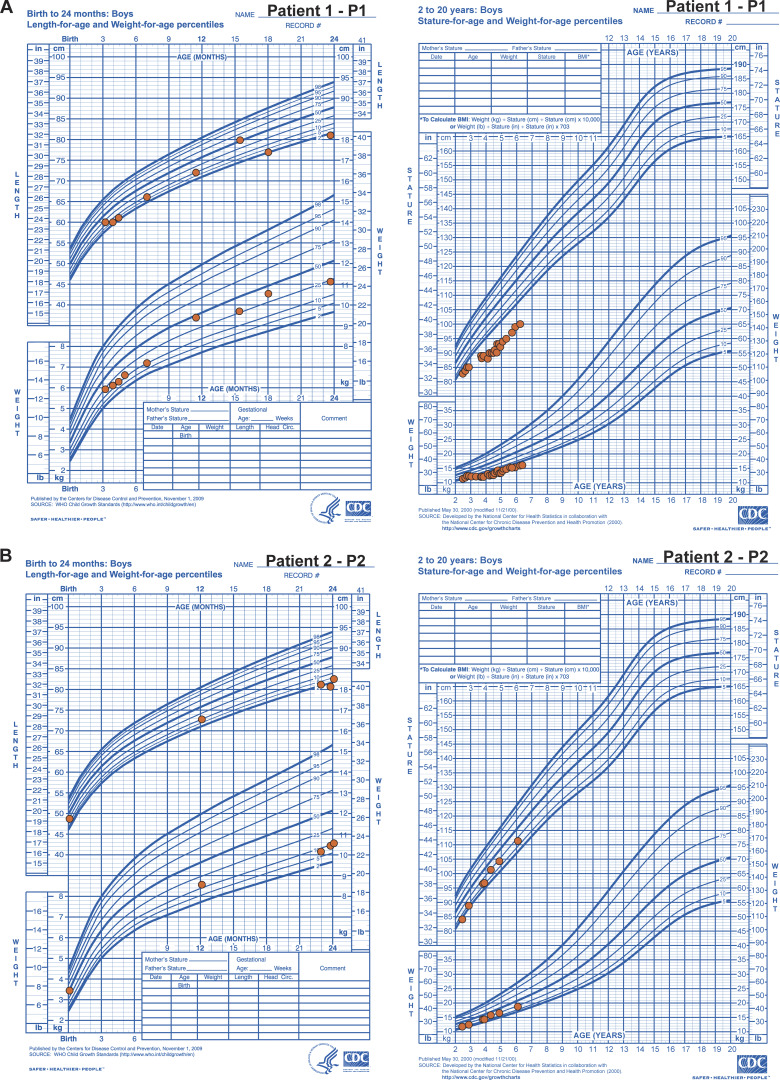
**Growth charts for patients with biallelic *OSMR* deficiency.** **(A)** P1 and **(B)** P2.

The 10 patients presented with similar phenotypes consistent with an underlying PAD (Fig. 2, A–C). Specifically, they exhibited severe, widespread, treatment-resistant atopic dermatitis (Fig. 2 D), which began in their first month of life, combined with atopic blood biomarkers of peripheral blood eosinophilia (Fig. 2 B) and markedly elevated serum IgE (Fig. 2 C). Some patients also presented with failure-to-thrive (Fig. S1). P1 began treatment with dupilumab, a biologic inhibitor targeting IL-4 and IL-13 signaling, at 4.5 years of age, resulting in a significant improvement in skin disease (Fig. 2 E), and P6 and P10 have also recently initiated treatment with this therapy. Individual patient summaries are provided in Table S1. Clinical immunophenotyping of patients with *OSMR* deficiency was essentially unremarkable and did not reveal a consistent immunologic signature (Table S2).

### Patient *OSMR* variants lead to a lack of OSMRβ cell surface expression

**Figure 3.**
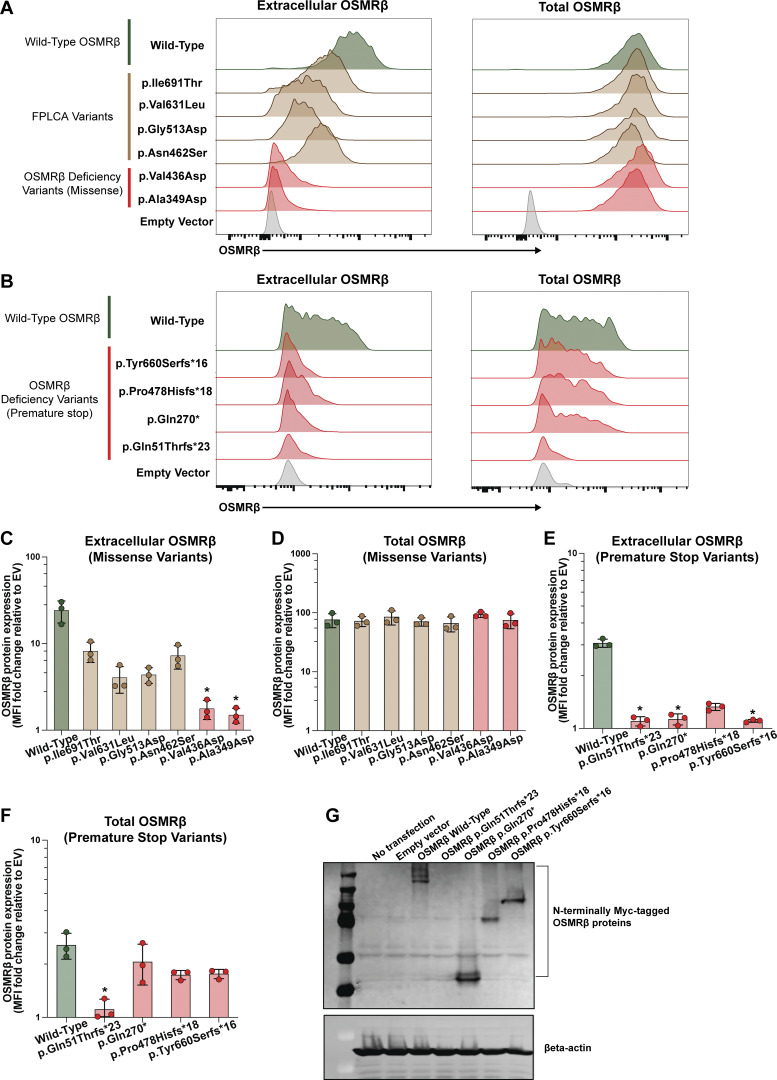
***OSMR* LOF variants lead to a lack of OSMRβ cell surface expression in HEK293 cells. (A)** Extracellular and total OSMRβ expression were quantified in HEK293 cells transfected with WT OSMRβ, OSMRβ missense variants, (p.Ala349Asp and p.Val436Asp), or FPLCA variants (p.Asn462Ser, p.Gly513Asp, p.Val631Leu, and p.Ile691Thr) using flow cytometry. OSMRβ expression was quantified in GFP^+^ cells; *n* = 3. **(B)** Extracellular and total OSMRβ expression were quantified in HEK293 cells transfected with either WT OSMRβ or OSMRβ variants that lead to a frameshift and premature stop (p.Gln51Thrfs*23, p.Gln270*, p.Pro478Hisfs*18, and p.Tyr660Serfs*16) using flow cytometry. OSMRβ expression was quantified in PE-OSMRβ^+^ cells instead of GFP^+^ cells as the variant results in a truncated protein, preventing translation of the C-terminal GFP; *n* = 3. **(C)** Quantification of A, extracellular OSMRβ expression; *n* = 3. **(D)** Quantification of A, total OSMRβ expression; *n* = 3. **(E)** Quantification of B, extracellular OSMRβ expression; *n* = 3. **(F)** Quantification of B, total OSMRβ expression; *n* = 3. **(G)** Immunoblot visualizing OSMRβ expression in HEK293 cells transfected with WT OSMRβ or the following OSMRβ variants, p.Gln51Thrfs*23, p.Gln270*, p.Pro478Hisfs*18, and p.Tyr660Serfs*16; *n* = 3. Statistical comparisons between OSMRβ WT and OSMRβ variants were performed using a Kruskal–Wallis test followed by Dunn’s multiple comparisons test. Asterisks indicate statistical significance (P < 0.05). Source data are available for this figure: SourceData F3.

To assess the functional impact of the p.Ala349Asp, p.Val436Asp, p.Gln51Thrfs*23, p.Gln270*, p.Pro478Hisfs*18, and p.Tyr660Serfs*16 OSMRβ variants, we modeled them in HEK293 cells. We selected HEK293 cells as our model system due to their low endogenous OSMRβ expression (35) (Fig. 3, A and B). As controls, in parallel, we tested wild-type (WT) OSMRβ and four OSMRβ missense variants reported in association with FPLCA (26, 27, 30, 31). WT OSMRβ and OSMRβ missense variants tagged with green-fluorescence protein (GFP) at the C terminus were overexpressed in HEK293 cells. Extracellular and total OSMRβ levels were assessed by antibody staining in the absence or presence of permeabilization, respectively, followed by flow cytometric analysis. Using this system, we demonstrated significantly decreased cell surface expression of the p.Ala349Asp and p.Val436Asp OSMRβ variant constructs compared to WT OSMRβ (P < 0.05) (Fig. 3, A and C). Cell surface expression was intermediate in cells transfected with FPLCA variants (p.Asn462Ser, p.Gly513Asp, p.Val631Leu, and p.Ile691Thr). Notably, total OSMRβ protein expression was indistinguishable between all groups, as shown by intracellular flow cytometry staining (Fig. 3, A and D).

**Figure S2.**
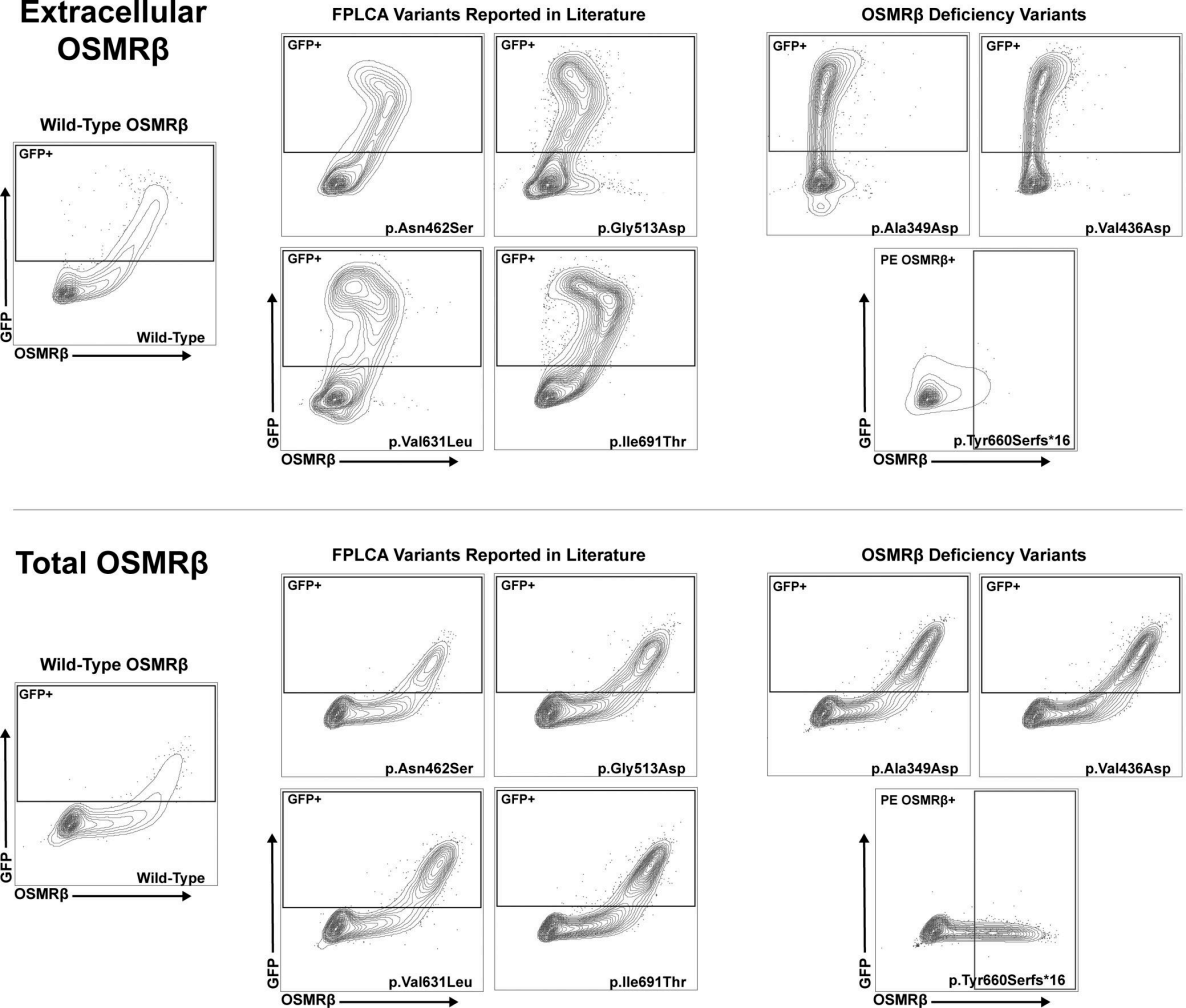
**Contour plots demonstrating loss of cell surface OSMRβ expression in HEK293 cells.** Extracellular and total OSMRβ expression were quantified in HEK293 cells transfected with WT OSMRβ, OSMRβ LOF variants (p.Ala349Asp, p.Val436Asp, p.Gln51Thrfs*23, p.Gln270*, p.Pro478Hisfs*18, and p.Tyr660Serfs*16), or FPLCA variants (p.Asn462Ser, p.Gly513Asp, p.Val631Leu, and p.Ile691Thr) using flow cytometry.

We modified the experimental approach to assess the patient premature termination codon *OSMR* variants (Fig. 3, E–G). To detect potential truncated protein products, we introduced an N-terminal Myc tag into OSMR constructs carrying variants predicted to generate premature termination codons. Immunoblotting showed that the p.Gln270*, p.Pro478Hisfs*18, and p.Tyr660Serfs*16 variants produce truncated but stable OSMRβ proteins, whereas the p.Gln51Thrfs*23 variant did not yield detectable protein (Fig. 3 G). These truncated proteins lack the transmembrane domain of OSMRβ and would therefore be predicted to fail to localize to the cell surface (Fig. 1 B). However, because the p.Tyr660Serfs*16 variant occurs in exon 14, which comprises 174 nucleotides, we studied potential in-frame skipping of exon 14 and preservation of the transmembrane domain. Assessing membrane localization, flow cytometric analysis demonstrated that the p.Gln270*, p.Pro478Hisfs*18, and p.Tyr660Serfs*16 variants result in truncated OSMRβ proteins that localize predominantly to the intracellular compartment, characterized by high total OSMRβ expression and minimal extracellular OSMRβ expression, with no detectable signal from the C-terminal GFP tag (Fig. 3, B, E and F; and Fig. S2; shown for p.Tyr660Serfs*16). The truncated variants retain the N-terminal signal peptide required for endoplasmic reticulum (ER) targeting but fail to traffic to the cell surface (36), suggesting retention in the ER. Flow cytometry similarly failed to detect the p.Gln51Thrfs*23 variant, consistent with the immunoblotting results.

Together, these data support a LOF mechanism for the missense (p.Ala349Asp, p.Val436Asp) and truncating (p.Gln270*, p.Pro478Hisfs*18, and p.Tyr660Serfs*16) OSMRβ variants, whereas the p.Gln51Thrfs*23 variant results in a loss of protein expression. Despite distinct molecular lesions, all variants display markedly reduced cell surface expression of OSMRβ relative to WT and FPLCA variants, consistent with impaired receptor availability at the plasma membrane and consequent disruption of OSMRβ-dependent signaling.

### OSMRβ cell surface expression is reduced in patient primary fibroblasts

**Figure 4.**
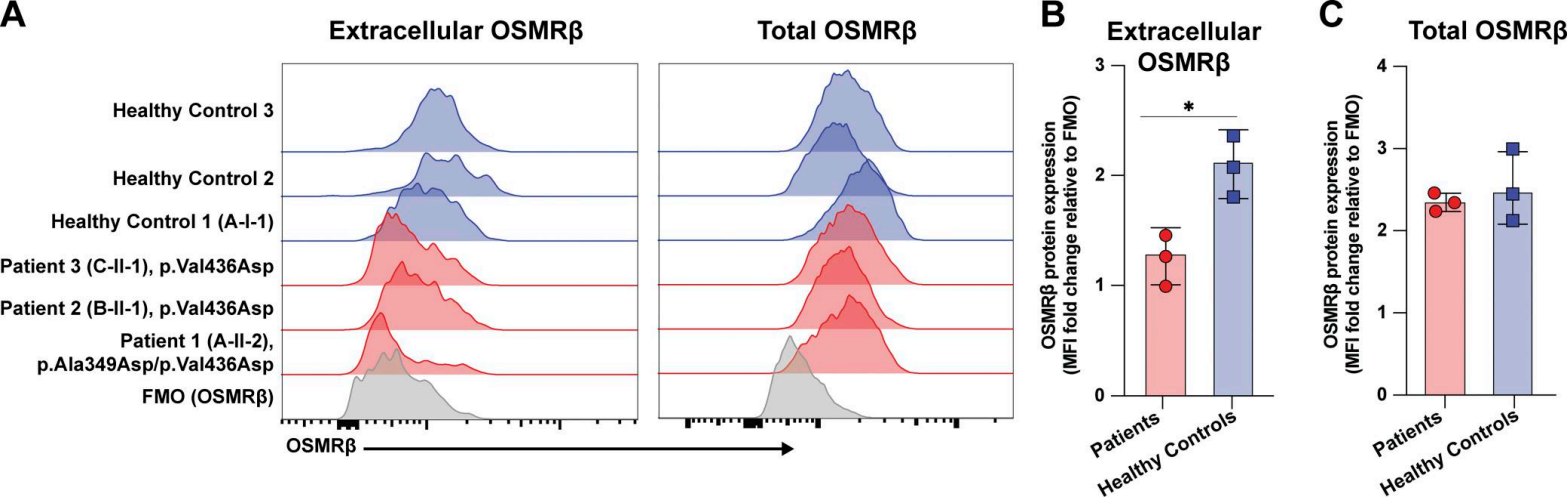
***OSMR* variants lead to decreased OSMRβ cell surface expression in primary fibroblasts. (A)** Extracellular and total OSMRβ expression were quantified in primary fibroblasts from P1, P2, and P3 (unable to obtain fibroblasts from P4 through P10) and three healthy controls using flow cytometry; *n* = 3. **(B)** Quantification of A, extracellular OSMRβ expression; *n* = 3. **(C)** Quantification of A, total OSMRβ expression; *n* = 3. Statistical comparisons based on unpaired *t* test. Asterisks denote P values: * < 0.05. FMO, fluorescence minus one.

To validate the findings from the in vitro HEK293 cell experiments, we quantified OSMRβ expression in primary fibroblasts from P1, P2, and P3 alongside three healthy controls (HCs); primary samples from P4 through P10 were not available. Cell surface expression was significantly lower in the primary patient fibroblasts compared to HCs (P = 0.02) (Fig. 4, A and B), whereas total OSMRβ expression was similar between patients and controls (Fig. 4, A and C). Notably, the cell surface OSMRβ expression in the healthy heterozygous mother of P1 (Family A-I-1) was comparable to the other two HCs, further reinforcing the autosomal recessive inheritance pattern of disease (Fig. 4 A).

### Population-level genomic evidence further supports pathogenicity of *OSMR* variants

**Figure 5.**
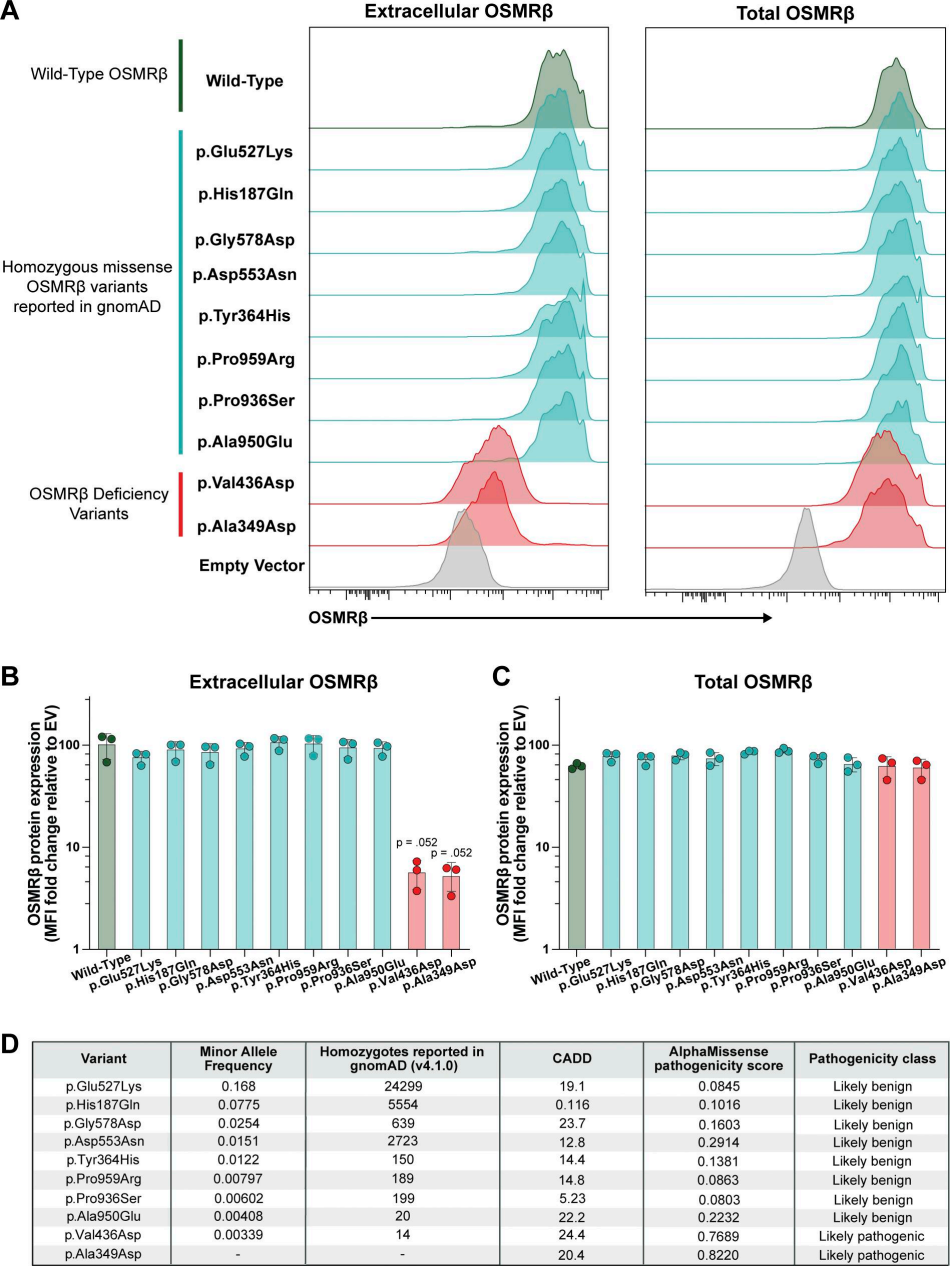
**Homozygous missense *OSMR* population variants show normal cell surface expression of OSMRβ in HEK293 cells and are predicted to be benign. (A)** Extracellular and total OSMRβ expression was quantified in HEK293 cells transfected with WT OSMRβ, OSMRβ disease-associated variants, (p.Ala349Asp and p.Val436Asp), or homozygous OSMRβ population variants reported in gnomAD with a MAF greater than the p.Val436Asp variant (p.Glu527Lys, p.His187Gln, p.Gly578Asp, p.Asp553Asn, p.Tyr364His, p.Pro959Arg, p.Pro936Ser, and p.Ala950Glu) using flow cytometry. OSMRβ expression was quantified in GFP^+^ cells; *n* = 3. **(B)** Quantification of A, extracellular OSMRβ expression; *n* = 3. **(C)** Quantification of A, total OSMRβ expression; *n* = 3. Statistical comparisons between OSMRβ WT and OSMRβ variants were performed using a Kruskal–Wallis test followed by Dunn’s multiple comparisons test. **(D)** MAFs, CADD scores, and AlphaMissense pathogenicity scores for OSMRβ population variants and OSMRβ disease-associated variants.

Leveraging population-level genomic data to assess pathogenicity, we examined the p.Val436Asp variant, which is reported in gnomAD (v4.1.0) with 14 homozygous individuals, including nine from the UK Biobank (UKB). The relatively high minor allele frequency (MAF = 0.00339) and the presence of multiple homozygotes in an unselected population might ordinarily argue against a classical IEI. However, available UKB clinical data suggest enrichment of allergic disease or cutaneous features among individuals homozygous for p.Val436Asp, including elevated peripheral eosinophil counts or percentages (3/9) and documented allergic or dermatologic diagnoses in several individuals. These findings should be interpreted cautiously given the small sample size and likely incomplete clinical penetrance (Table S3). To place this observation in context, we systematically evaluated all homozygous coding OSMRβ variants reported in gnomAD with MAFs exceeding that of p.Val436Asp. In contrast to the patients’ OSMRβ variants, these higher-frequency population variants localized to the cell surface at levels comparable to WT OSMRβ and were predicted to be benign by AlphaMissense (Fig. 5, A–D), highlighting the discriminatory value of AlphaMissense predictions for normal cell surface expression of OSMRβ (37).

### Biallelic LOF variants in *OSMR* impair signaling through the OSM/OSMRβ axis

**Figure 6.**
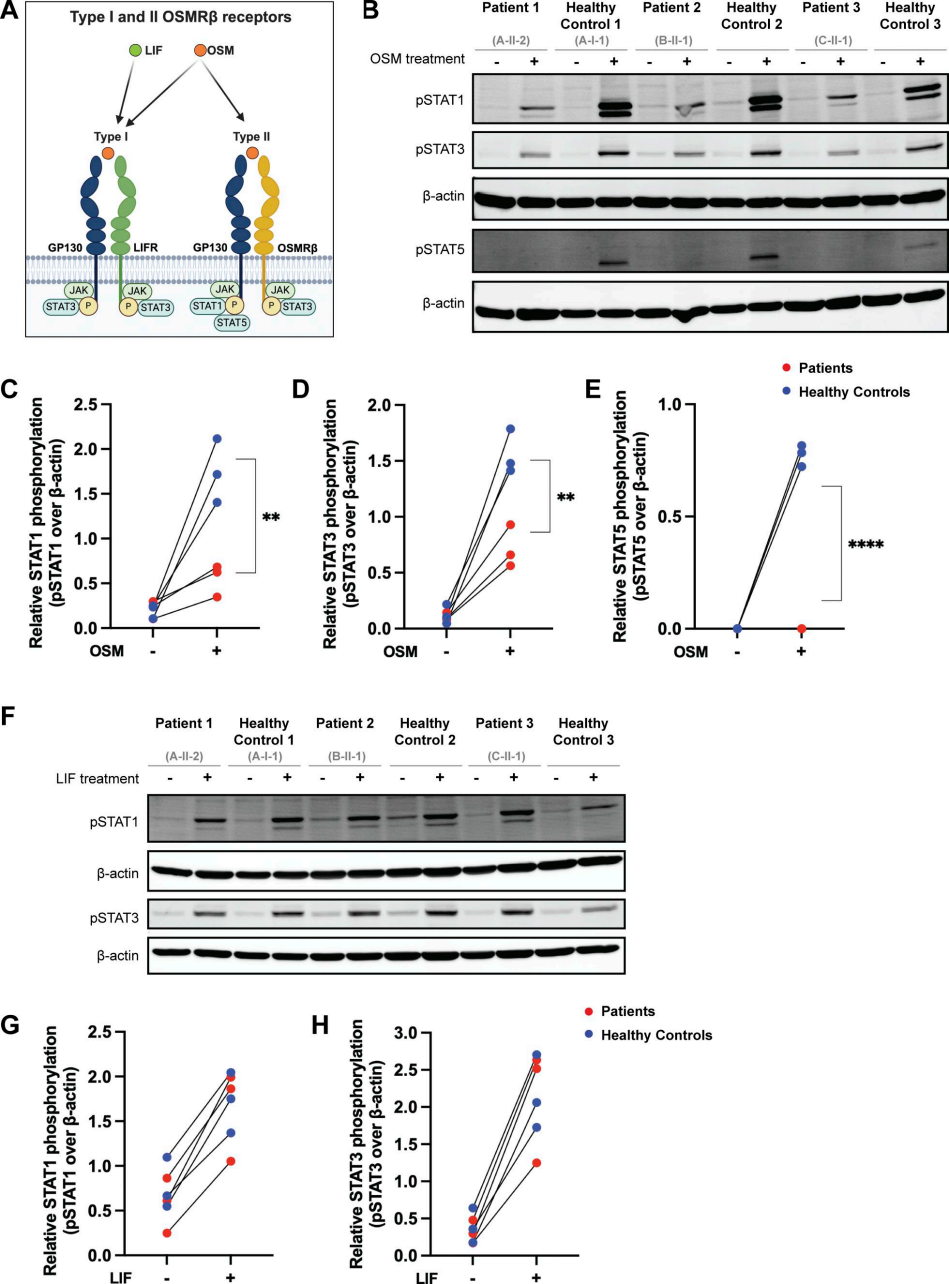
***S*elective impairment of STAT signaling through the OSM/OSMRβ axis in primary fibroblasts. (A)** Schematic illustrating the OSM/OSMRβ axis. **(B)** Immunoblot in primary fibroblasts from P1, P2, and P3 (unable to obtain fibroblasts from P4 though P10) and three HCs for pSTAT1, pSTAT3, and pSTAT5 before and after treatment with OSM (100 ng/ml for 15 min); *n* = 3. **(C–E)** Quantification of the immunoblot in B for (C) pSTAT1, (D) pSTAT3, and (E) pSTAT5. Patients are in red and healthy controls are in blue. Statistical comparisons based on unpaired *t* test. Asterisks denote P values: **P < 0.01; ****P < 0.0001. **(F)** Immunoblot in primary fibroblasts from P1, P2, and P3 and three HCs for pSTAT1 and pSTAT3 before and after treatment with LIF (100 ng/ml for 15 min); *n* = 3. **(G and H)** Quantification of the immunoblot in F for (G) pSTAT1 and (H) pSTAT3. Patients are in red, and healthy controls are in blue. Source data are available for this figure: SourceData F6.

Given that cell surface OSMRβ expression was low in the overexpression system and on primary cells, we hypothesized that this would likely impair signaling through the OSM/OSMRβ axis (Fig. 6 A). We tested this hypothesis using primary fibroblasts from P1, P2, and P3. Activation of STAT5 after stimulation with OSM is exclusively mediated by the OSM type II receptor (OSMRβ/GP130) and was absent in patient cells. Similarly, patient cells displayed significantly decreased activation of STAT1 and STAT3 after OSM stimulation, with the residual activation likely reflecting signaling through the OSM type I receptor (LIFR/GP130) (Fig. 6, B–E). Notably, heterozygous control fibroblasts from a parent (Family A-I-1) were indistinguishable from the other HC fibroblasts, indicating that impairment of the OSM/OSMRβ axis requires biallelic LOF.

**Figure S3.**
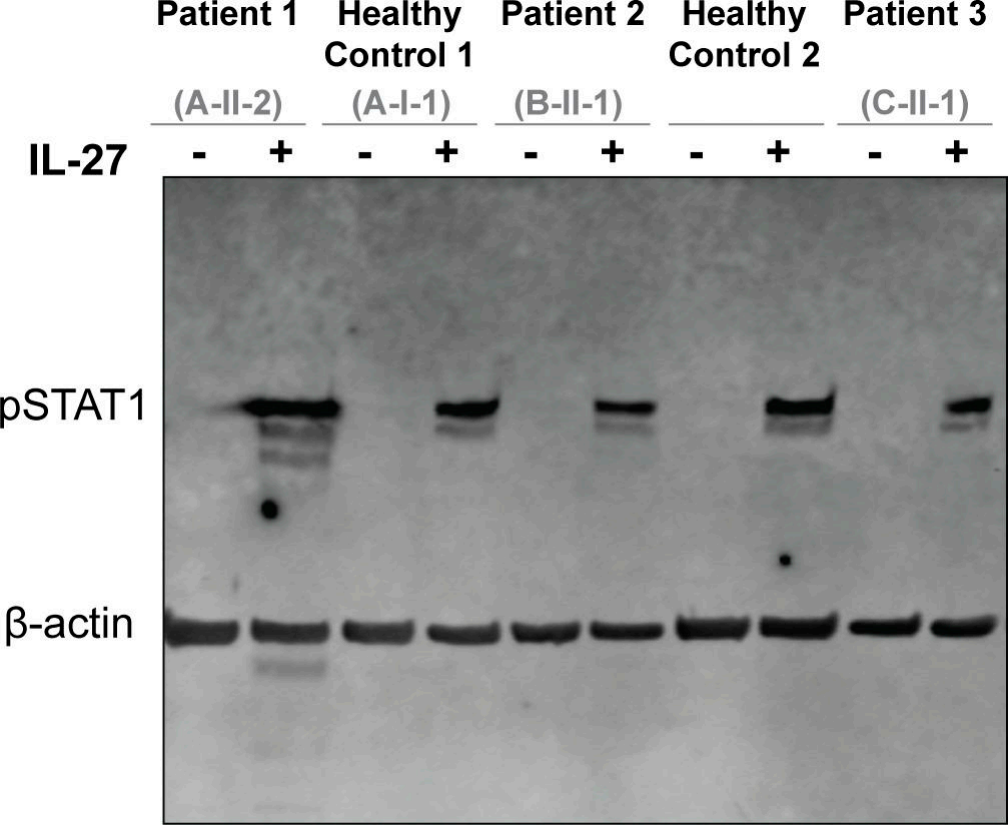
**IL-27 stimulation, which signals through the IL-27Rα/GP130 complex, induced comparable STAT1 phosphorylation in fibroblasts from patients and healthy controls.** Immunoblot of primary fibroblasts from P1, P2, and P3 alongside HCs showing pSTAT1 before and after treatment with IL-27 (200 ng/ml for 15 min); *n* = 3. Source data are available for this figure: SourceData FS3.

To establish the specificity of the signaling defect, we assessed signaling through other GP130-containing cytokine receptor complexes in primary patient fibroblasts. In contrast to OSM stimulation, both leukemia inhibitory factor (LIF)– and IL-27–mediated STAT activation were preserved. Stimulation with LIF, which signals through the LIFR/GP130 complex, induced comparable downstream responses in fibroblasts from patients and HCs (Fig. 6, F–H) (38). Similarly, stimulation with IL-27, which signals through the IL-27Rα/GP130 complex, induced comparable STAT1 phosphorylation in fibroblasts from patients and HCs (Fig. S3). Together, these data demonstrate that biallelic LOF variants in *OSMR* selectively impair OSMRβ-dependent signaling while preserving signaling through other GP130-associated receptor complexes.

### Expression of WT OSMRβ restores OSM signaling in patient fibroblasts

**Figure 7.**
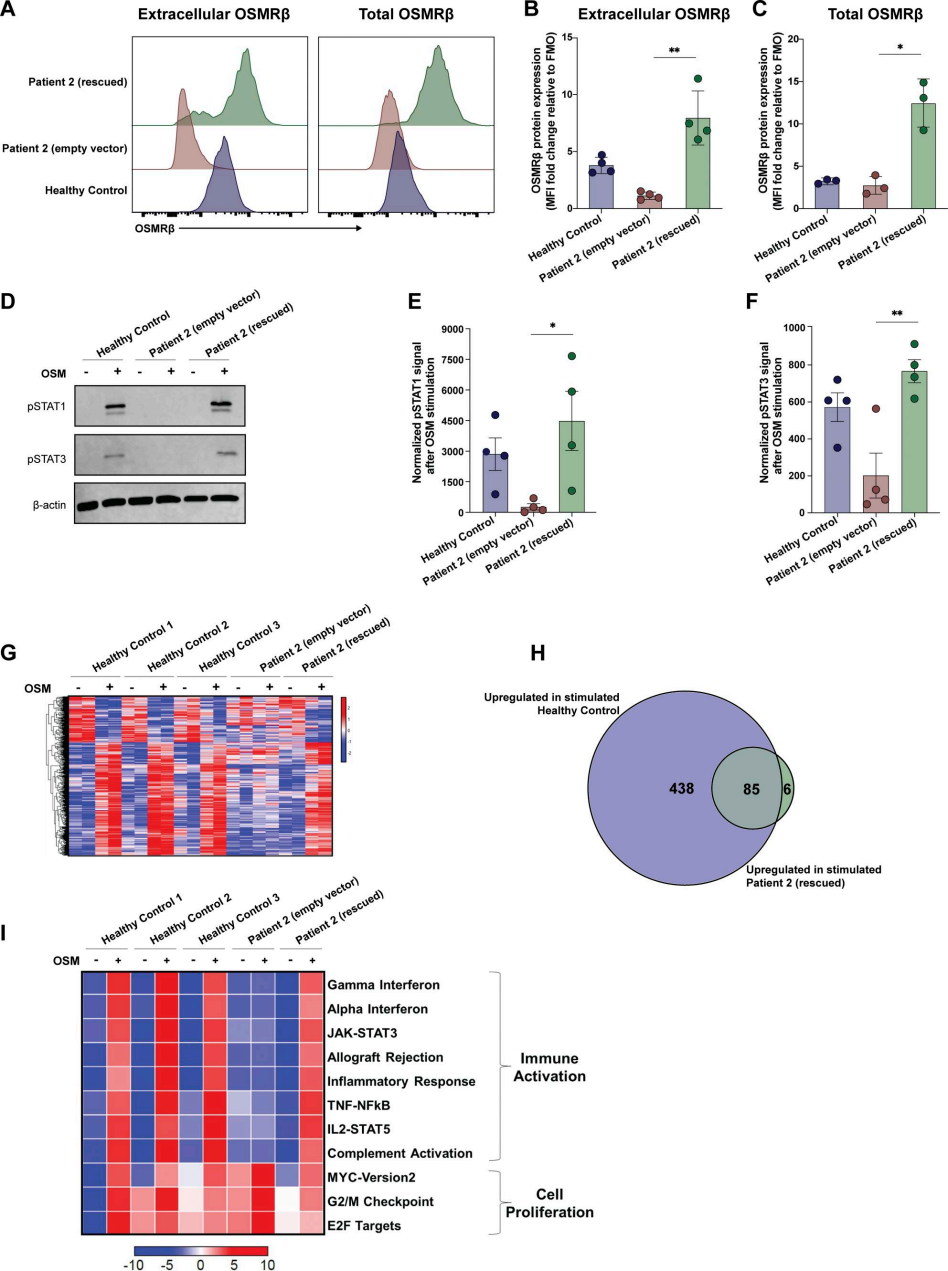
**WT *OSMR* restores the OSM/OSMRβ axis and STAT signaling in primary fibroblasts. (A)** Extracellular and total OSMRβ expression were quantified in primary fibroblasts from P2 that were transduced with WT *OSMR* (rescued) or an EV and compared with primary fibroblasts from an HC using flow cytometry; *n* = 3. **(B)** Quantification of A, extracellular OSMRβ expression; *n* = 3. **(C)** Quantification of A, total OSMRβ expression; *n* = 3. **(D)** Immunoblot in primary fibroblasts from P2 that were transduced with WT *OSMR* or an EV and compared to primary fibroblasts from an HC for pSTAT1 and pSTAT3 before and after treatment with OSM (100 ng/ml for 15 min); *n* = 4. **(E and F)** Quantification of the immunoblot in D for (E) pSTAT1 and (F) pSTAT3. **(G)** Heatmap signatures of differentially expressed genes comparing P2 fibroblasts (either transduced with WT *OSMR* or an EV) against three HCs before and after stimulation with OSM (100 ng/ml for 15 min). **(H)** Overlap in significantly upregulated genes upon OSM stimulation in HC cells (blue) and P2 rescued cells (green) as shown through a Venn diagram. **(I)** GSEA of significantly enriched immune pathways from the MSigDB Hallmark in P2 (either transduced with WT *OSMR* or an EV) against three HCs before and after stimulation with OSM (100 ng/ml for 15 min). Heatmap is normalized across the rows and shown as relative expression of sample level enrichment scores. Statistical comparisons based on the Kruskal–Wallis H test. Asterisks denote P values: *< 0.05, **< 0.01. Source data are available for this figure: SourceData F7.

**Figure S4.**
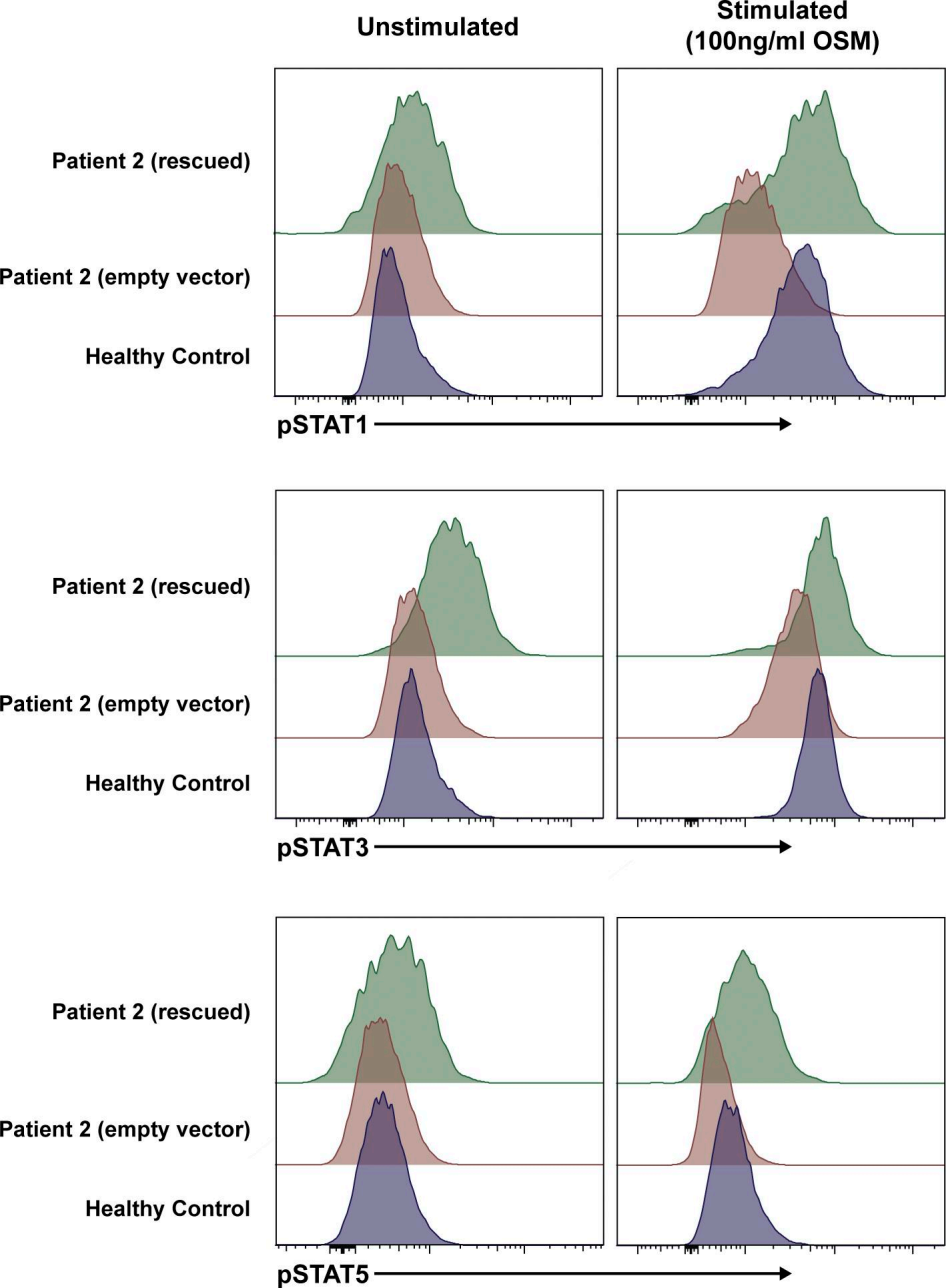
**WT *OSMR* restores STAT signaling in primary fibroblasts.** Phosphorylation of STAT1, STAT3, and STAT5 was quantified in primary fibroblasts from P2 that were transduced with WT *OSMR* or an EV and compared to primary fibroblasts from an HC before and after treatment with OSM (100 ng/ml for 15 min); *n* = 3.

To establish a causal relationship between the genotype and cellular phenotype (39), we used a lentiviral approach to re-express WT OSMRβ in primary dermal fibroblasts of P2. Lentiviral transduction rescued cell surface expression of OSMRβ (Fig. 7, A–C). Lentiviral transduction also rescued signaling through the OSM axis, as measured by pSTAT1 and pSTAT3 (Fig. 7, D–F and Fig. S4). Finally, transcriptomic analysis confirmed a profound defect in OSM-induced gene regulation in patient fibroblasts that was largely corrected by re-expression of WT OSMRβ (Fig. 7, G and H). In HC fibroblasts, OSM stimulation induced 523 significantly upregulated and 211 downregulated genes. In contrast, P2 fibroblasts transduced with empty vector (EV) showed no significant differential gene expression following OSM treatment. Re-expression of WT OSMRβ in P2 fibroblasts restored OSM responsiveness, resulting in 91 significantly upregulated genes and no downregulated genes. Notably, 85 of these 91 genes overlapped with OSM-induced genes in HC fibroblasts, demonstrating substantial rescue of the transcriptional program downstream of OSMRβ signaling (Fig. 7 H).

Differential pathway activation using gene set enrichment analysis (GSEA) showed significant enrichment in pathways for immune activation and cell proliferation in HCs after stimulation with OSM (Fig. 7 I). Consistent with established links between STAT1 activation and interferon signaling (40, 41), we found that OSM stimulation led to strong activation of interferon activation pathways (e.g., HC: Gamma_IFN normalized enrichment score 2.71, adj P value < 0.001), as well as STAT3, STAT5, and MYC pathway activation (Fig. 7 I and Table S4). These pathways were either reduced or absent in patient fibroblasts (Fig. 7 I and Table S4). Reinforcing causality, lentiviral rescue of P2 fibroblasts with WT-*OSMR* restored signaling in all immune-activated pathways downstream of OSMRβ. Collectively, these findings establish the role of OSMRβ in OSM-mediated immune activation of fibroblasts.

## Discussion

In this study, we define a new human PAD caused by germline biallelic LOF variants in *OSMR* in 10 patients with early-onset severe atopic disease. These variants all led to a lack of cell surface expression of OSMRβ protein and decreased OSM-mediated STAT1, STAT3, and STAT5 activation and phosphorylation. As this study includes only 10 affected individuals, defining the full clinical spectrum of this disorder and elucidating all the underlying mechanisms will require identification of additional patients and further mechanistic studies.

**Figure 8.**
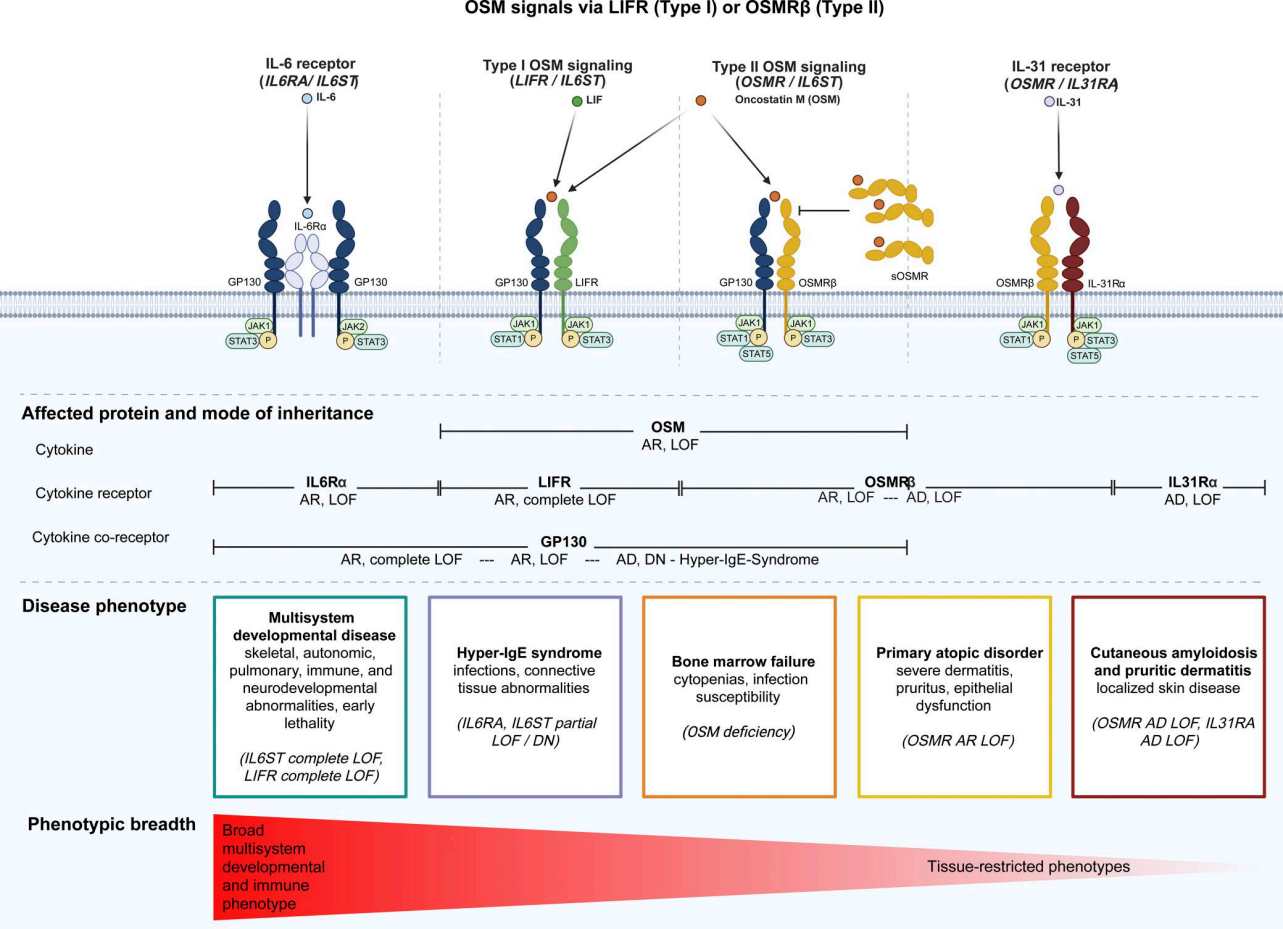
**Hierarchical organization of monogenic disorders in the IL-6 cytokine family pathway.** Schematic of IL-6 family receptor complexes and associated human genetic disorders. OSM signals via LIFR/GP130 (type I) and OSMRβ/GP130 (type II), while IL-31 signals through OSMRβ/IL-31RA and IL-6 through IL-6Rα/GP130. The lower panel maps cytokine, receptor, and co-receptor defects to inheritance patterns and clinical phenotypes. Complete *IL6ST* and *LIFR* deficiency cause severe multisystem developmental disease; partial or dominant-negative *IL6ST* variants result in hyper-IgE–like phenotypes; *OSM* deficiency causes bone marrow failure; biallelic *OSMR* LOF, defined in this study, causes a PAD; and dominant *OSMR* or *IL31RA* variants cause cutaneous amyloidosis. The gradient reflects a proposed hierarchy of phenotypic breadth according to the position of the affected gene within the signaling cascade. AD, autosomal dominant; AR, autosomal recessive; DN, dominant negative; GP130, glycoprotein 130; JAK, Janus Kinase; LIFR, leukemia inhibitory factor receptor; sOSMR, soluble OSMRβ; STAT, signal transducer and transactivator.

The identification of biallelic *OSMR* deficiency fills a previously unassigned disease node within the human IL-6 family cytokine signaling pathway (Fig. 8). At the most proximal level, biallelic complete LOF variants in *IL6ST* (GP130) disrupt signaling by multiple IL-6 family cytokines causing a severe multisystem disorder, termed extended Stüve–Wiedemann syndrome, encompassing lethal cardiopulmonary defects, skeletal abnormalities, autonomic dysfunction and additional variable clinical features such as congenital thrombocytopenia, eczematoid dermatitis, renal abnormalities, and defective acute-phase response (42). In contrast, loss of *LIFR* causes Stüve–Wiedemann syndrome, characterized by congenital skeletal dysplasia and autonomic instability (43). More restricted, predominantly immune phenotypes resembling *STAT3*-related hyper-IgE syndrome are observed in individuals with autosomal recessive hypomorphic or autosomal dominant-negative *IL6ST* variants (44, 45). Selective disruption of the IL-11 signaling axis further illustrates the functional specialization within this pathway, as defects in *IL11RA* or IL-11–selective variants in *IL6ST* produce predominantly craniofacial and skeletal phenotypes, including craniosynostosis and dental abnormalities (46, 47). More recently, biallelic deficiency of the OSM ligand itself has been shown to cause bone marrow failure, revealing a nonredundant role for OSM in human hematopoiesis (11, 12). In a parallel branch of this pathway, defects in *IL31RA* disrupt signaling through the IL-31 receptor complex and are associated with pruritic skin disease, including cutaneous amyloidosis in humans (31, 48), while experimental models further support a role for IL-31 signaling in pruritus, epithelial inflammation, and type 2 immune responses (10, 49, 50). Our findings establish *OSMR* LOF as a distinct receptor-level disorder that selectively impairs OSM-dependent signaling while preserving broader IL-6 family pathways, thereby defining a PAD that is mechanistically and phenotypically intermediate between ligand-level defects and disruptions of the shared co-receptor or down-stream JAK/STAT signaling. Together, these observations refine genotype–phenotype relationships across the IL-6 family and support a hierarchical model in which disease severity are shaped by both the position of the affected gene within the signaling cascade and the breadth of downstream cytokine responses that are lost.

Population genetics provide another layer of evidence supporting the involvement *OSMR* genetic variation in human immunity. Several independent genome-wide association studies have found that polymorphisms in *OSMR* associate with multiple chronic inflammatory conditions, including Crohn’s disease (51, 52, 53), ulcerative colitis (51, 52, 53), psoriasis (53), ankylosing spondylitis (53), and sclerosing cholangitis (53). Recently, microbial genome-wide interaction studies also found that interactions between single-nucleotide polymorphisms in the *OSMR* gene and early-life exposures, such as breastfeeding, are associated with gut microbiota alterations linked to asthma and atopy (54).

An important distinction we establish in this study is the difference between biallelic autosomal recessive *OSMR* deficiency and FPLCA. Whilst there is overlap between the conditions, there are key differences. The most notable are that FPLCA is typically an autosomal dominant disorder with disease limited to the skin and onset in adolescence or adulthood, while the patients we describe all had the onset of severe atopic dermatitis from infancy accompanied by other manifestations of their allergic diathesis. Notably, none of the *OSMR* variants identified in this study have previously been associated with FPLCA, despite multiple clinical reports implicating *OSMR* in this condition. In addition, heterozygous carriers of the LOF alleles identified here did not report skin disease, supporting a recessive disease mechanism distinct from the dominant inheritance of missense variants observed in FPLCA. Moreover, the amyloid deposits in the superficial dermis, which are a defining feature of FPLCA, were lacking in the skin biopsies of patients with biallelic *OSMR* deficiency (30). Functional data emphasize the differences between FPLCA and biallelic *OSMR* deficiency. We show that FPLCA variants lead to intermediate levels of cell surface expression of OSMRβ, which contrasts with the absence of cell surface expression we find with the autosomal recessive *OSMR* LOF variants. Our result is consistent with a previous study of OSMRβ expression by microscopy that found no differences in the localization of FPLCA-associated OSMRβ variants when compared to OSMRβ^WT^ (55). Ultimately, the distinct features and potential overlap between autosomal dominant FPLCA and autosomal recessive *OSMR* deficiency will be fully defined through the diagnosis of additional affected individuals.

Despite known species differences, the *Osmr*^*−/−*^ mouse accurately models the epithelial and signaling defects observed in human biallelic *OSMR* deficiency. In both settings, loss of OSMRβ results in impaired OSM-dependent STAT signaling and prominent abnormalities in epithelial homeostasis, with *Osmr*^*−/−*^ mice displaying increased epidermal thickness and our patients manifesting early-onset severe atopic dermatitis and barrier dysfunction (55). This concordance supports a conserved role for OSMRβ in postnatal skin biology across species. In contrast, systemic phenotypes reported in *Osmr*^*−/−*^ mice, including hematopoietic and skeletal abnormalities, were not observed in our cohort, likely reflecting species-specific ligand–receptor usage and context-dependent activation of OSM signaling pathways (38, 56, 57, 58, 59, 60). Together, these findings indicate that while murine models incompletely capture the full human phenotype, they accurately model the epithelial and signaling defects that define biallelic *OSMR* deficiency as a PAD.

Our data suggest that biallelic OSMR deficiency may exhibit variable expressivity and likely incomplete clinical penetrance. For example, in family G, although all three homozygous individuals were affected, the severity of their atopic dermatitis varied substantially, ranging from particularly severe to more manageable disease. Similarly, analysis of UKB data indicated that among nine individuals homozygous for *OSMR* c.1307T>A (p.Val436Asp), at least two had no documented abnormalities in eosinophil counts, allergic manifestations, or skin phenotypes, although phenotyping in UKB is incomplete. Independent clinical observations further support the pathogenic potential of this allele. Andersen et al. recently described a patient homozygous for *OSMR* c.1307T>A (p.Val436Asp) who presented with elevated IgE levels, atopic dermatitis, chronic pulmonary aspergillosis, and bone fractures (32).

To place these findings in the broader population genetic context, we systematically assessed cell surface expression of all homozygous coding *OSMR* variants reported in gnomAD with MAFs exceeding that of p.Val436Asp. In contrast to the patients’ *OSMR* variants, these higher-frequency population variants localized to the cell surface at levels comparable to WT OSMRβ and were predicted to be benign by AlphaMissense. These results indicate that homozygosity for *OSMR* missense variants does not uniformly result in impaired receptor surface expression and underscore the importance of integrating structural context, functional assays, and computational prediction tools such as AlphaMissense when interpreting *OSMR* variants.

Together, these observations suggest that biallelic *OSMR* LOF may represent an under-recognized genetic contributor to severe allergic disease. There is precedent for this pattern in other genetically mediated allergic disorders. For example, relatively common LOF variants in *FLG* impair epithelial barrier function and are strongly associated with atopic dermatitis and downstream allergic disease (61). More recently, *JAK1* variants with mild-to-moderate GOF activity have been linked to relatively common presentations of inflammatory, allergic, and/or autoimmune disease (62). These examples highlight how variants with incomplete penetrance can nevertheless make substantial contributions to disease susceptibility when present in appropriate genetic contexts.

In conclusion, we identify biallelic LOF variants in *OSMR* in 10 patients from seven unrelated families, establishing *OSMR* deficiency as a new PAD and revealing an essential role for OSMRβ signaling in maintaining immune and barrier homeostasis in humans. With >50 genes now recognized to cause PADs (5), clinicians should consider inclusive next-generation sequencing approaches when evaluating patients with severe or atypical allergic disease, as timely molecular diagnosis can guide targeted therapy and improve clinical outcomes. More broadly, these findings highlight how the study of rare monogenic PADs can illuminate fundamental pathways governing allergic inflammation and immune homeostasis.

## Materials and methods

### Ethical considerations

All study participants and/or their parents/guardians provided written informed consent for sequencing, data analysis, and publication of findings and images. Research study protocols were approved by The University of British Columbia Clinical Research Ethics Board (H15-0064), the NIH Institutional Review Board (NCT00852943, NCT01164241), or King Faisal Specialist Hospital and Research Centre (2241173).

Participants were included in our research study if they harbored biallelic variants in the *OSMR* gene and had clinical manifestations consistent with atopic disease. Participants ranged in age from infancy to 74 years of age. No participant attrition occurred during the study, and all enrolled participants were included in the analyses.

### Identification of *OSMR* variants using whole-exome sequencing

Whole-exome sequencing was performed on genomic DNA from patients as either trio-based or singleton analyses. For singleton cases, segregation of candidate *OSMR* variants was evaluated in available family members by Sanger sequencing. The identified *OSMR* variants were predicted to be damaging and segregated with disease and thus were selected for further analysis (63, 64, 65).

### UKB

Data for this study were extracted under UKB application number 103789. Data processing was performed using the UKB Research Analysis Platform. Whole-exome sequencing data from 469,835 participants (data field 23158: Population-level exome OQFE variants, PLINK format – final exome release) were analyzed to identify homozygous carriers of the *OSMR* variant c.1307T>A, p.Val436Asp (NM_003999.3; rs34324145; SNV 5-38917567-T-A [GRCh38]). Homozygous genotypes were extracted using JupyterLab on the UKB DNANexus platform. Basic demographic information (sex, age at inclusion) and phenotype data (ICD-10–coded diagnoses, data field 41270; eosinophil count and percentage at first blood draw) were obtained using the cohort browser tool on the same platform.

### Generation of *OSMR* variant plasmids

Plasmids used for transfection studies contained full-length *OSMR* cDNA in a pCMV6-AC-GFP vector with a C-terminal GFP tag (Cat#: RG216943; OriGene Technologies). To generate *OSMR* LOF variants (c.1046C>A, c.1307T>A, c.1979_1980delAC, c.150dup, c.808C>T, c.1433del), homozygous population database variants (c.561T>G, c.1090T>C, c.1579G>A, c.1657G>A, c.1733G>A, c.2806C>T, c.2849C>A, c.2876C>G), and other literature-reported FPLCA variants (c.2072T>C, c.1891G>T, c.1538G>A, c.1385A>G), a Q5 site-directed mutagenesis kit (Cat#: E0554S; New England Biolabs) was used according to the manufacturer’s recommendations, with primer pairs noted in Table S5.

Plasmids used for immunoblotting transfection experiments were based on the same construct containing full-length *OSMR* cDNA cloned into a pCMV6-AC-GFP vector with a C-terminal GFP tag. A Myc tag was inserted between residues 27 and 28, immediately downstream of the signal peptide cleavage site (36), thereby generating an N-terminally Myc-tagged mature OSMRβ protein. The following primers were used to add the Myc tag as well as linker regions: forward primer 5′-GGC​GGC​GGC​GGC​AGC​GAG​CAG​AAA​CTC​ATC​TCA​GAA​GAG​GAT​CTG​GGC​GGC​GGC​GGC​AGC​GAA​CGT​TTA​CCA​TTG​ACT​CCT​GTA​TCA​C-3′ and reverse primer 5′-AGC​CAA​GAC​TTC​ACT​CTG​GTA​AGT​CC-3′. To generate *OSMR* frameshift and premature stop variants, c.150dup, c.808C>T, c.1433del, and c.1979_1980delAC, a Q5 site-directed mutagenesis kit (Cat#: E0554S; New England Biolabs) was used according to the manufacturer’s recommendations, with primer pairs noted in Table S5.

To generate lentivirus vectors, WT OSMRβ from the above plasmids was cloned into a Lenti vector with a C-terminal GFP tag (Cat#: PS100071; OriGene Technologies) using EcoRI-HF (Cat#: R3101; New England Biolabs) and NotI-HF (Cat#: R3189; New England Biolabs). The plasmids were packaged using third-generation packaging plasmids and transfected into HEK293T cells (RRID:CVCL_0063). This was also done for an empty Lenti vector, which was used as a control. Culture media was collected, centrifuged, filtered, concentrated, and stored at −80°C before use.

All *OSMR* variant plasmids were confirmed by Sanger sequencing and purified from 10-β competent *Escherichia coli* using a QIAprep Spin Miniprep Kit (Cat#: 27104; Qiagen).

### Isolation and culture of primary dermal fibroblasts

Primary fibroblasts were isolated from a non-lesional skin punch biopsy (P1 and P2) or a surgical skin biopsy (P3). The skin was excised from the underlying connective tissue and placed in separate wells of a 6-well plate in DMEM (GE Healthcare) supplemented with 10% heat-inactivated FBS (Gibco, Life Technologies), 2 mM L-glutamine (HyClone, Thermo Fisher Scientific), 1 mM sodium pyruvate (Gibco, Life Technologies), and 1× Antibiotic-Antimycotic (Gibco, Thermo Fisher Scientific) for 3 wk at 37°C, or until a confluent monolayer of fibroblasts had formed. Primary fibroblasts were then lifted and frozen for future assays. Fibroblasts were Sanger sequenced to confirm the genotype of the cells before use in experiments.

### Transient and stable expression of *OSMR* variants

Transient expression of *OSMR* variants in HEK293 (RRID:CVCL_0045) cells was accomplished using a Lipofectamine 3000 kit (Thermo Fisher Scientific) according to the manufacturer’s recommendation. Briefly, HEK293 cells were seeded at 8.0 × 10^5^ cells/well in a 6-well plate in 1.5 ml of DMEM supplemented with 10% FBS and incubated for 24 h at 37°C. Cells were transfected with 2.5 μg of plasmid DNA using P3000 and Lipofectamine 3000 reagents and harvested after 24 h. HEK293 cells were tested for mycoplasma contamination every 2 mo and were authenticated by short tandem repeat profiling by the manufacturer.

Stable expression of OSMRβ in primary dermal fibroblasts was accomplished using the lentivirus approach as previously described (66, 67). Briefly, primary dermal fibroblasts were infected with lentiviral particles in the presence of 5 μg/ml polybrene (Sigma-Aldrich), cultured, and expanded in DMEM supplemented with 10% FBS (Gibco, Life Technologies). Expanded cells were sorted on GFP expression using a BD FACS Aria (BD Biosciences) cell sorter.

### Cell surface and intracellular flow cytometry

OSMRβ expression and phospho-STAT detection were quantified using flow cytometry in transfected and primary cells. For cell surface OSMRβ expression, transfected HEK293 cells or primary fibroblasts were lifted and stained with *OSMR* PE (Cat#:12-1303-42; Thermo Fisher Scientific; RRID:AB_1633423) for 20 min. Expression was measured in GFP^+^ HEK293 cells or primary fibroblasts on an LSRII flow cytometer (BD Biosciences) and analyzed using FlowJo software (BD Biosciences; RRID:SCR_008520). For total OSMRβ and phospho-STAT detection, cells were fixed using BD Cytofix (Cat#: 554655; BD Biosciences) for 20 min at 4°C and permeabilized using Perm III for 30 min on ice (Cat#: 558050; BD Biosciences). The cells were then stained with OSMRβ PE, or with pSTAT1 BV421 (Cat#: 562985; BD Biosciences; RRID:AB_2737932), pSTAT3 PECF594 (Cat#: 562673; BD Biosciences; RRID:AB_2737714), and pSTAT5 PeCy7 (Cat#: 560117; BD Biosciences; RRID:AB_1645546). OSMRβ expression was measured in GFP^+^ HEK293 cells that were transfected with the p.His187Gln, p.Ala349Asp, p.Tyr364His, p.Val436Asp, p.Asn462Ser, p.Gly513Asp, p.Glu527Lys, p.Asp553Asn, p.Gly578Asp, p.Val631Leu, p.Ile691Thr, pPro936Ser, p.Ala950Glu, or p.Pro959Arg variants. OSMRβ expression was measured in PE^+^ HEK293 cells that were transfected with p.Gln51Thrfs*23, p.Gln270*, p.Pro478Hisfs*18, or p.Tyr660Serfs*16 variants (because these variants results in a truncated protein and thus the C-terminal GFP would not be translated). OSMRβ expression was measured in primary fibroblasts as well. Cells were analyzed on an LSRII flow cytometer (BD Biosciences), and flow cytometry data were analyzed using FlowJo software (BD Biosciences).

### Immunoblotting

Immunoblotting was conducted as previously described (68, 69) to assess the phosphorylation status of STAT1, STAT3, and STAT5 in primary fibroblasts stimulated with OSM, LIF, or IL-27 and to quantify OSMRβ expression in HEK293 cells. Primary fibroblasts and HEK293 cells were cultured in DMEM. Cells were harvested in chilled radioimmunoprecipitation assay (RIPA) buffer (Cat# 89901; Thermo Fisher Scientific) supplemented with HALT protease and phosphatase inhibitor cocktail (Cat# 87786; Thermo Fisher Scientific) and then lysed for 15 min on ice. Cell lysates were separated by 12% SDS-polyacrylamide gel electrophoresis and transferred onto polyvinylidene fluoride (PVDF) membranes (Cat# IPFL00010; Immobilon-FL; MilliporeSigma). Membranes were blocked with 5% BSA in TRIS-buffered saline with Tween-20 for an hour and then incubated with primary antibodies in blocking buffer overnight at 4°C. The next day, membranes were washed and incubated with secondary antibodies for 1 h at room temperature and then imaged using a LI-COR Odyssey infrared scanner (LI-COR Biosciences).

The primary antibodies used were the following: pSTAT1 (Cat#: 9167; Cell Signaling Technologies; RRID:AB_561284), pSTAT3 (Cat#: 9138; Cell Signaling Technologies; RRID:AB_331262), pSTAT5 (Cat#: 9356; Cell Signaling Technologies; RRID:AB_331263), Myc-tag (Cat#: 2276; Cell Signaling Technologies; RRID:AB_331783), and β-actin (Cat#: 3700; Cell Signaling Technologies; RRID:AB_2242334). The secondary antibodies used were the following: goat anti-rabbit IgG DyLight 800 conjugated (Cat#: 611-145-002-0.5; Rockland Immunochemicals; RRID:AB_11183542) and goat anti-mouse IgG IRDye 680RD (Cat#: 926-68070; LI-COR; RRID:AB_10956588).

### RNA sequencing

RNA was extracted, sequenced, and then preprocessed as previously described (68, 70, 71). Expression data were then normalized to reads between samples using the edgeR package in R (R Foundation; RRID:SCR_012802). Normalized counts were filtered to remove low counts using the filterByExpr function in edgeR (72). Differential expression between unstimulated and stimulated samples for healthy control fibroblasts, P2-EV fibroblasts, and P2-WT-*OSMR* was accomplished using Limma (73) (RRID:SCR_010943). Differentially expressed genes were defined as those with a fold change >1.25 and an Benjamini–Hochberg adjusted P value < 0.05.

Pathway analysis was done by first performing GSEA with 1,000 permutations using the Molecular Signatures Database Hallmark module. The signal-to-noise ratio was used for gene ranking and the obtained normalized enrichment scores, and P values were further adjusted using the Benjamini–Hochberg method. Pathways with an adjusted P value <0.05 were considered significant. Leading-edge genes from significant pathways between HC unstimulated fibroblasts and stimulated fibroblasts were identified. Expression levels of these genes were then determined in each of the three groups (healthy control fibroblasts, P2-EV, and P2-WT-*OSMR*). Sample level enrichment analysis scores were computed as previously described (74). Briefly, z-scores were computed for gene sets of interest for each sample. The mean expression levels of significant genes were compared to the expression of 1,000 random gene sets of the same size. The difference between observed and expected mean expression was then calculated and represented on heatmaps.

### Online supplemental material

Fig S1 contains growth charts for P1 and P2, demonstrating failure to thrive. Fig S2 contains contour plots showing that the *OSMR* variants lead to a lack of OSMRβ cell surface expression in HEK293 cells. Fig S3 shows a representative immunoblot of patient and control fibroblasts stimulated with IL-27, indicating that signaling remains intact. Fig S4 contains additional data showing that WT *OSMR* transduction restores STAT signaling in primary fibroblasts. Table S1 summarizes the clinical characteristics of the 10 *OSMR*-deficient patients. Table S2 presents available clinical immunophenotyping data from five patients against published reference ranges (75). Table S3 summarizes phenotypical data of UKB listed homozygous carriers of the p.Val436Asp variant. Table S4 lists significantly enriched pathways identified in OSM-stimulated patient and healthy control fibroblasts. Table S5 provides the primer sequences used for site-directed mutagenesis of the *OSMR *gene.

## Supplementary Material

Table S1 shows clinical characteristics of patients with biallelic *OSMR* variants.

Table S2 shows T, B, and NK cell quantification and serum immunoglobulin values in patients with *OSMR* deficiency.

Table S3 shows clinical data from the UKB for individuals who are homozygous for the *OSMR* p.Val436Asp variant.

Table S4 shows list of pathways that are significantly activated in patient and healthy control fibroblasts after stimulation with OSM.

Table S5 shows list of primers used for site-directed mutagenesis in the *OSMR* gene.

SourceData F3 is the source file for Fig. 3.

SourceData F6 is the source file for Fig. 6.

SourceData F7 is the source file for Fig. 7.

SourceData FS3 is the source file for Fig. S3.

## Data Availability

Data are deposited at Gene Expression Omnibus (RRID:SCR_005012) with the following identifier: GSE303962.
