# Supplementary material for: Human germline biallelic loss-of-function OSMR variants cause severe allergic disease
Source: J Hum Immun. 2026 May 28;2(4):e20260067. doi: 10.70962/jhi.20260067 (PMC13218299; doi:10.70962/jhi.20260067)
Supplement: Table S1 — shows clinical characteristics of patients with biallelic OSMR variants. [file jhi_20260067_tables1.docx]

Supplementary Table 1. **Clinical characteristics of patients with biallelic *OSMR* variants.**

| Patient | P1 | P2 | P3 | P4 | P5 | P6 | P7 | P8 | P9 | P10 |
| --- | --- | --- | --- | --- | --- | --- | --- | --- | --- | --- |
| Age | 6 y | 10 y | 74 y | 9 mo | 18 mo | 8 y | 4 y | 17 y | 15 y | 20 mo |
| Consanguinity | None | None | Unknown | Yes | Yes | Yes | Yes | Yes | Yes | Yes |
| Parents | Healthy | Healthy | Unknown | Healthy | Healthy | Healthy | Healthy | Healthy | Healthy | Healthy |
| Disease manifestation | | | | | | | | | | |
| Atopic dermatitis | Generalized,  severe | Extensive,  severe | Severe since infancy, seasonal worsening | Severe since birth | Severe since birth | Severe since childhood | Severe since infancy | Rather mild since infancy | Face eczema since infancy | Therapy refractory, severe since birth (face and scalp) |
| Allergies | Multiple IgE-mediated food allergies, sensitization to dust mite and dog’s hair | Sensitization to house dust mites, cat, and dog dander | None | Sensitization to milk, peanut, and soybean | Cow’s milk | Multiple food allergies | None | Allergic rhinoconjunctivitis, sensitization to house dust mite | Allergic rhinoconjunctivitis, sensitization to house dust mite | Sensitization to egg white, cow’s milk protein, banana, peanut, soybean, house dust mite, mold, tree, and grass pollen |
| Eosinophilic gastrointestinal disease | Esophagitis | None | None | Generalized | None | None | None | None | None | None |
| Angioedema | None | Yes | None | None | None | None | None | None | None | None |
| Failure-to-thrive | Yes | Yes | Unknown | Poor weight gain | None | None | None | None | None | None |
| Lymphoproliferation | Significant generalized lymphadenopathy | None | None | None | None | None | None | None | None | None |
| Recurrent infections | Respiratory viral, rec. otitis media | Mild upper respiratory infections | None | None | None | None | None | None | None | None |
| Other symptoms | Adrenal insufficiency, optic nerve papilledema, and intracranial hypertension | None | None | Mild developmental delay with delayed speech | None | None | None | None | None | None |
| Biopsies and histological findings | | | | | | | | | | |
| Bone marrow | Trilineage hematopoiesis | None | None | None | None | None | None | None | None | None |
| Intestinal | Mild epithelial hyperplasia / spongiosis, increased eosinophils in the esophagus, focally increased eosinophils in colonic mucosa | None | None | None | None | None | None | None | None | None |
| Lymph node | Reactive lymphoid hyperplasia | None | None | None | None | None | None | None | None | None |
| Skin | Epidermal acanthosis, spongiosis, superficial perivascular inflammatory infiltrate with numerous eosinophils and overlying seropurulent crusting | None | None | Spongiosis, neutrophilic crusting in epidermis, lymphocytic infiltration in upper dermis | None | None | None | None | None | None |
| Treatments | | | | | | | | | | |
| Topical | Steroids | Steroids | None | Steroids | Steroids | None | None | Pimecrolimus | Steroids | Steroids, pimecrolimus |
| Systemic | Dupilumab | Antibiotics, occasionally systemic corticosteroids | Oral antihistamine (loratadine) | Baricitinib | None | Dupilumab | None | None | None | Dupilumab |

Abbreviations: mo: months; y: years
