# Supplementary material for: Human germline biallelic loss-of-function OSMR variants cause severe allergic disease
Source: J Hum Immun. 2026 May 28;2(4):e20260067. doi: 10.70962/jhi.20260067 (PMC13218299; doi:10.70962/jhi.20260067)
Supplement: Table S2 — shows T, B, and NK cell quantification and serum immunoglobulin values in patients with OSMR deficiency. [file jhi_20260067_tables2.docx]

Supplementary Table 2. **T- , B-, and NK-cell quantification and serum immunoglobulin values in patients with *OSMR* deficiency**

Reference ranges from Shearer et al. are provided in square brackets (Shearer et al., 2003). Values falling outside the reference range are indicated in bold. NA=not available.

| Patient | P1 | P2 | P10 | P9 | P8 |
| --- | --- | --- | --- | --- | --- |
| Age at testing | 6-year-old | 1.5-year-old | 1.5-year-old | 15-year-old | 17-year-old |
| Total lymphocytes (cells/µl) | 3550 [1900-3700] | 8750 [3600-8900] | 5700 [3600-8900] | 2830 [1400-3300] | 2770 [1400-3300] |
| CD3+ lymphocytes (cells/µl) | 1988 [1200-2600] | 4460 [2100-6200] | 4845 [2100-6200] | **2408** [1000-2200] | **2410** [1000-2200] |
| CD3+ CD4+ lymphocytes (cells/µl) | 1065 [650-1500] | 3300 [1300-3400] | 3265 [1300-3400] | **1722** [530-1300] | **1576** [530-1300] |
| CD3+ CD8+ lymphocytes (cells/µl) | 781 [490-1300] | 900 [620-2000] | 1390 [620-2000] | 602 [330-920] | 733 [330-920] |
| CD19+ lymphocytes (cells/µL) | 639 [270-860] | **3030** [720-2600] | 708 [720-2600] | 156 [110-570] | 72 [110-570] |
| CD16+CD56+ lymphocytes (cells/µl) | **888** [100-480] | 270 [180-920] | NA | NA | NA |
| Serum IgG (g/L) | 11.4 [6.3-12.8] | 5.85 [3.5-12.1] | NA | NA | NA |
| Serum IgA (g/L) | **<0.05** [0.33-2.0] | 0.38 [0.44-1.1] | NA | NA | NA |
| Serum IgM (g/L) | 0.81 [0.48-2.1] | 0.92 [0.43-1.7] | NA | NA | NA |
