# Supplementary material for: Human germline biallelic loss-of-function OSMR variants cause severe allergic disease
Source: J Hum Immun. 2026 May 28;2(4):e20260067. doi: 10.70962/jhi.20260067 (PMC13218299; doi:10.70962/jhi.20260067)
Supplement: Table S3 — shows clinical data from the UKB for individuals who are homozygous for the OSMR p.Val436Asp variant. [file jhi_20260067_tables3.docx]

Supplementary Table 3. **Clinical data from the UK Biobank for individuals who are homozygous for the *OSMR* p.Val436Asp variant.**

Out of the nine individuals with available clinical data, seven presented with either a skin or atopic phenotype. One individual without any reported clinical data was excluded.

|  | Sex | Age | Eosinophil Count [cells/µL, normal=0-500] | Eosinophil Count [%, normal=0-5] | Allergic Manifestations | Skin Phenotype |
| --- | --- | --- | --- | --- | --- | --- |
| Individual 1 | m | 67 | 200 | 2.3 | not reported | **malignant melanoma; unspecified skin neoplasm (face)** |
| Individual 2 | m | 65 | n/a | n/a | **irritant contact dermatitis** | **malignant skin neoplasm** |
| Individual 3 | f | 42 | 200 | 4 | not reported | **benign skin neoplasm** |
| Individual 4 | f | 55 | 200 | 2.3 | not reported | not reported |
| Individual 5 | m | 62 | **770** | **12.02** | not reported | not reported |
| Individual 6 | f | 43 | **700** | **8.2** | **allergic rhinitis (pollen)** | not reported |
| Individual 7 | f | 60 | 100 | 1.9 | not reported | not reported |
| Individual 8 | m | 55 | 100 | 1.7 | not reported | **granulomatous skin disorder, chalazion** |
| Individual 9 | f | 68 | 410 | **5.82** | not reported | not reported |
