# Supplementary material for: Human germline biallelic loss-of-function OSMR variants cause severe allergic disease
Source: J Hum Immun. 2026 May 28;2(4):e20260067. doi: 10.70962/jhi.20260067 (PMC13218299; doi:10.70962/jhi.20260067)
Supplement: Table S4 — shows list of pathways that are significantly activated in patient and healthy control fibroblasts after stimulation with OSM. [file jhi_20260067_tables4.docx]

|  | **NES** | **p** | **q** | **NES** | **p** | **q** | **NES** | **p** | **q** |
| --- | --- | --- | --- | --- | --- | --- | --- | --- | --- |
| **Gamma_IFN** | 2.71 | <0.001 | <0.001 | 2.27 | <0.001 | <0.001 | 2.56 | <0.001 | <0.001 |
| **Alpha_IFN** | 2.61 | <0.001 | <0.001 | 2.13 | <0.001 | <0.001 | 2.52 | <0.001 | <0.001 |
| **JAK-STAT3** | 2.17 | <0.001 | <0.001 | 1.6 | 0.027 | 0.04 | 2.05 | <0.001 | <0.001 |
| **Allo_reject** | 2.15 | <0.001 | <0.001 | 1.53 | 0.014 | 0.059 | 2.07 | <0.001 | <0.001 |
| **Inflam_resp** | 2.11 | <0.001 | <0.001 | 1.45 | 0.028 | 0.092 | 2.11 | <0.001 | <0.001 |
| **TNF_NFkB** | 1.94 | <0.001 | <0.001 | -1.77 | <0.001 | 0.025 | 1.83 | <0.001 | 0.002 |
| **IL2-STAT5** | 1.88 | <0.001 | 0.001 | 1.43 | 0.021 | 0.099 | 1.86 | <0.001 | 0.001 |
| **Complement** | 1.8 | <0.001 | 0.001 | -1.19 | 0.119 | 0.337 | 1.77 | 0.001 | 0.004 |
| **MYC_V2** | 1.73 | 0.003 | 0.005 | 1.06 | 0.369 | 0.625 | 1.46 | 0.052 | 0.089 |
| **G2M** | 1.7 | <0.001 | 0.007 | 1.95 | <0.001 | 0.002 | 0.55 | 1 | 1 |
| **E2F** | 1.64 | <0.001 | 0.015 | 1.71 | <0.001 | 0.018 | -0.98 | 0.57 | 1 |

Supplementary Table 4. List of pathways that are significantly activated in patient and healthy control fibroblasts after stimulation with OSM.

EV = empty vector; HC = healthy control; NES = normalized enrichment scores.
