# Supplementary material for: Human germline biallelic loss-of-function OSMR variants cause severe allergic disease
Source: J Hum Immun. 2026 May 28;2(4):e20260067. doi: 10.70962/jhi.20260067 (PMC13218299; doi:10.70962/jhi.20260067)
Supplement: Table S5 — shows list of primers used for site-directed mutagenesis in the OSMR gene. [file jhi_20260067_tables5.docx]

Supplementary Table 5. List of primers used for site-directed mutagenesis in the *OSMR* gene. The site of nucleotide change is underlined and bolded in the forward primer sequence.

|  | Variant | Forward Primer | Reverse primer |
| --- | --- | --- | --- |
| **Literature reported FPLCA variants** | c.2072T>C; p.Ile691Thr (Arita et al., 2008) | 5’-AAATACAAAA**C**TGACAACCCG-3’ | 5’-GCAACATTCTGAACCATC-3’ |
|  | c.1891G>T; p.Val631Leu (Tanaka et al., 2010) | 5’-CAACCCTCAC**T**TGCTGGTGGA-3’ | 5’-TCTGAAGGAGCAAGTTCC-3’ |
|  | c.1538G>A; p.Gly513Asp (Lin et al., 2010) | 5’-AACAGTGTGG**A**TGCTTCTCCT-3’ | 5’-GTTGGCTATGACGCAGATT-3’ |
|  | c.1385A>G; p.Asn462Ser (Wali et al., 2015) | 5’-CTGCATGCCA**G**TGGAAAGATC-3’ | 5’-TTTTGATAATGGCTTCCAG-3’ |
| **Autosomal recessive LOF variants** | c.1046C>A; p.Ala349Asp | 5’-GCCACAAATG**A**CATCATGACC-3’ | 5’-ATTTACATTTTCAAAGTTGACACTAAAAG-3’ |
|  | c.1307T>A; p.Val436Asp | 5’-GCCCCTGATG**A**CTGGAGAATTG-3’ | 5’-CTCTGAGGGAGCAGCTTC-3’ |
|  | c.1979_1980delAC; p.Tyr660Serfs*16 | 5’-CATGTCTATCTGAAATCCAAG-3’ | 5’-ACCCTTGTATAAAACCAG-3’ |
|  | c.150dup; p.Gln51Thrfs*23 | 5’-ACAATGGACTGTCCACAAC-3’ | 5’-TAAGTGCAAACTCTGACG-3’ |
|  | c.808C>T; p.Gln270* | 5’-GTGGTCTAAATAACCTTCCCAAAGC-3’ | 5’-CCCAAGGCAGTGTCCGTC-3’ |
|  | c.1433del; p.Pro478Hisfs*18 | 5’- ATCCAGTTCAGAGCT-3’ | 5’-GTTTGTCTAGGTTTTCTACAA -3’ |
| **Autosomal recessive benign population variants** | c.561T>G; p.His187Gln | 5’- AACAGATTCAgGGAGAACAAC-3’ | 5’- TCCCTTCCAAATAACAGG -3’ |
|  | c.1090T>C; p.Tyr364His | 5’- TAATTTCACAcATTTGTGTCAGATTG -3’ | 5’- TTCCTTATGGAGTGCACC -3’ |
|  | c.1579G>A; p.Glu527Lys | 5’- TGCAGACCCCaAAAACAAAGAG -3’ | 5’- GAGATGACTATTACAGAAGC -3’ |
|  | c.1657G>A; p.Asp553Asn | 5’- CCAACCTGGAaATGTTATAGG -3’ | 5’- GGTTTCCAAGACAGAGAG -3’ |
|  | c.1733G>A; p.Gly578Asp | 5’- AAGAATGTAGaTCCCAATACCAC -3’ | 5’- CCACTGGAAATCACCGAG -3’ |
|  | c.2806C>T; p.Pro936Ser | 5’- CCCAACAAACtCAGTAGAGGC -3’ | 5’- TTTATACTCTGAACAGTGTGG -3’ |
|  | c.2849C>A; p.Ala950Glu | 5’- ATGCAAATGGaAGTCTCCCTG -3’ | 5’-CATGTCTATCTGAAATCCAAG-3’ |
|  | c.2876C>G; p.Pro959Arg | 5’- GCCTTGCCTCgCCCGACCGAG -3’ | 5’- AAGACGCAGGGAGACTGCC -3’ |
