# Supplementary figures and images for: Human germline biallelic loss-of-function OSMR variants cause severe allergic disease
Source: J Hum Immun. 2026 May 28;2(4):e20260067. doi: 10.70962/jhi.20260067 (PMC13218299; doi:10.70962/jhi.20260067)

# Source Data for Figure 3G

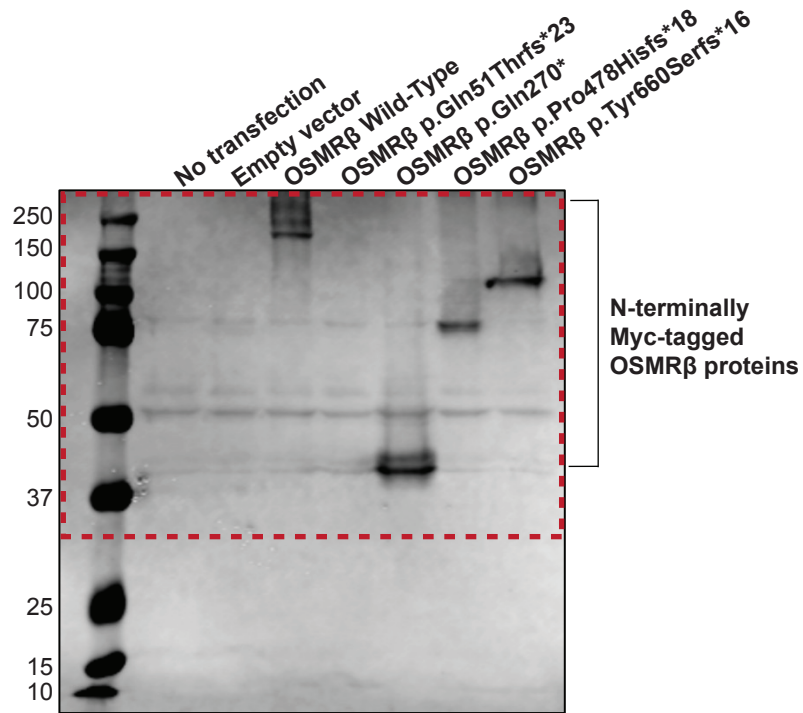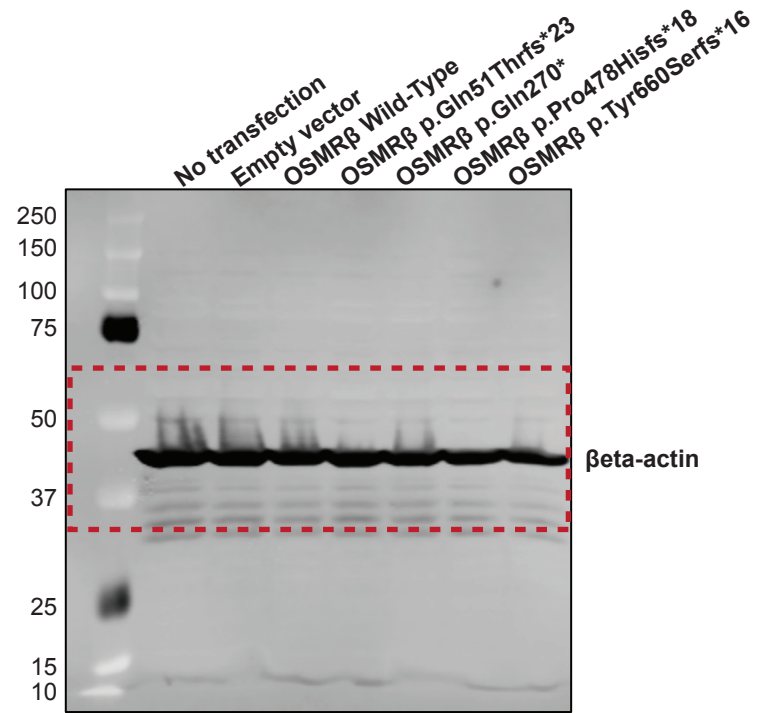

Supplement: SourceData F3 — is the source file for Fig. 3. [file jhi_20260067_sourcedataf3.pdf]

Source Data for Figure 6B

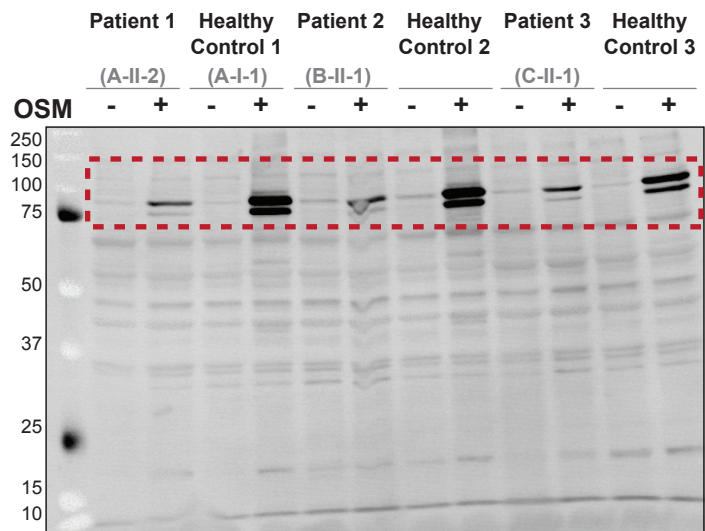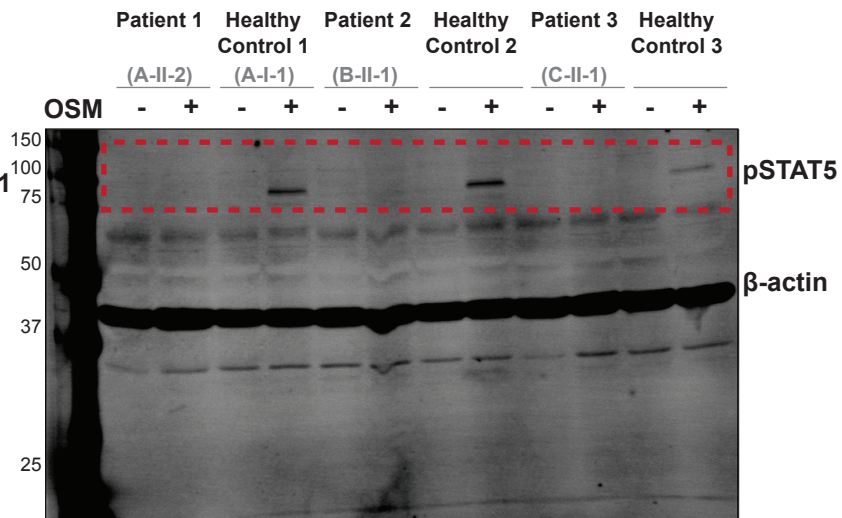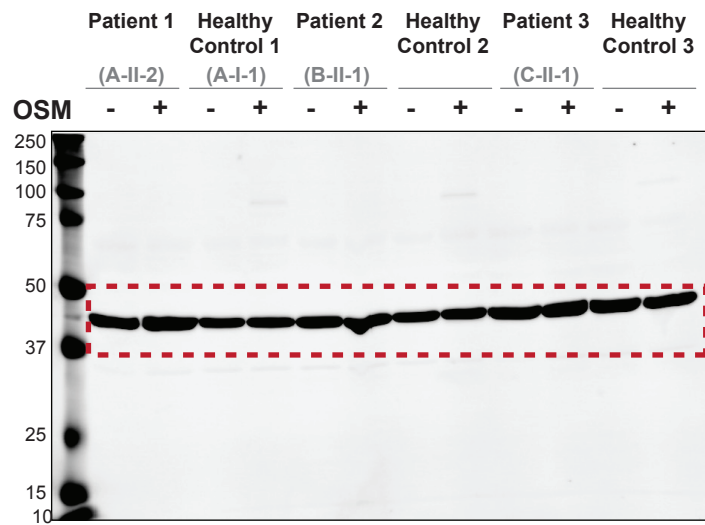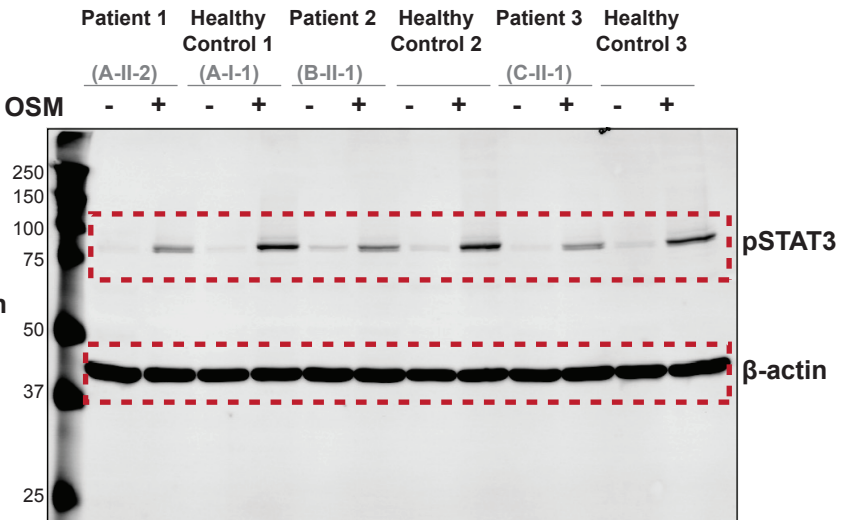

Source Data for Figure 6F

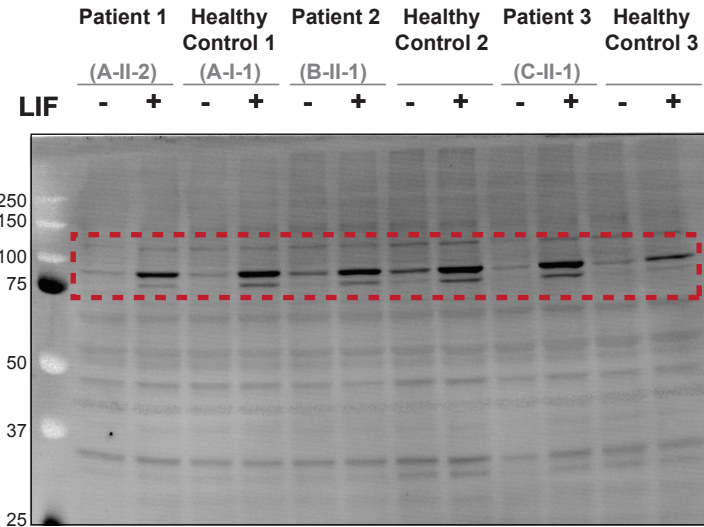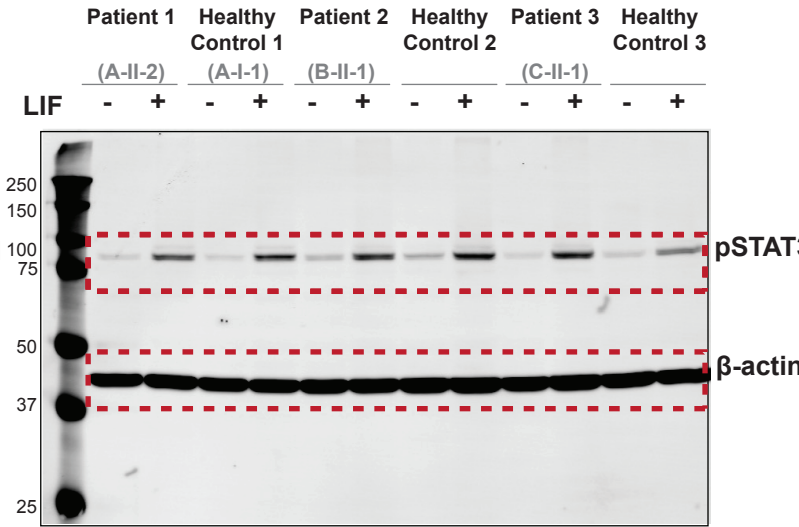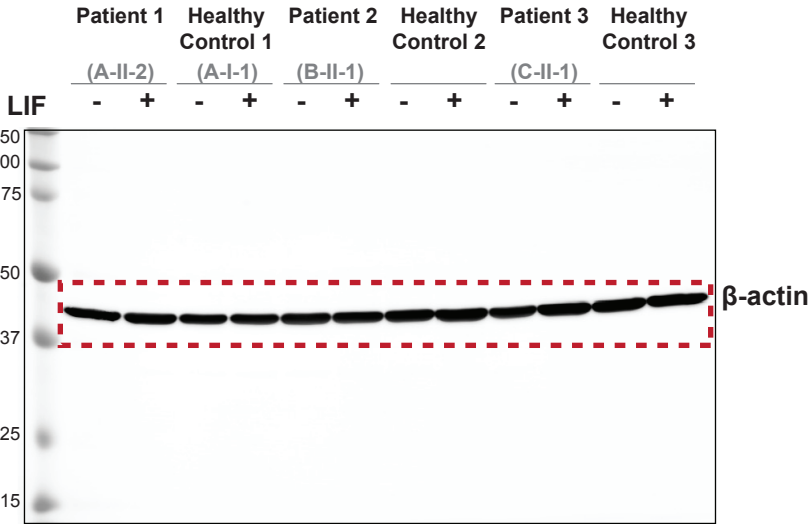

Supplement: SourceData F6 — is the source file for Fig. 6. [file jhi_20260067_sourcedataf6.pdf]

## Source Data for Figure 7D

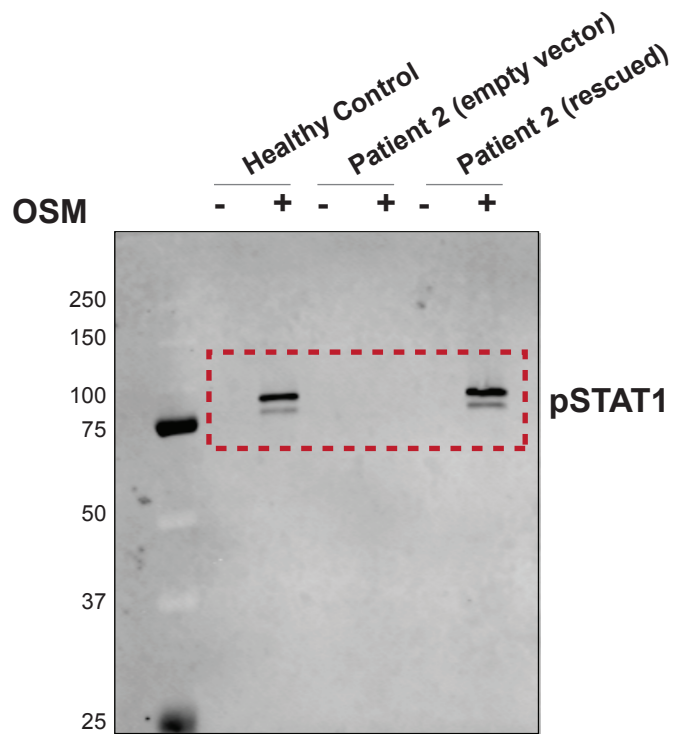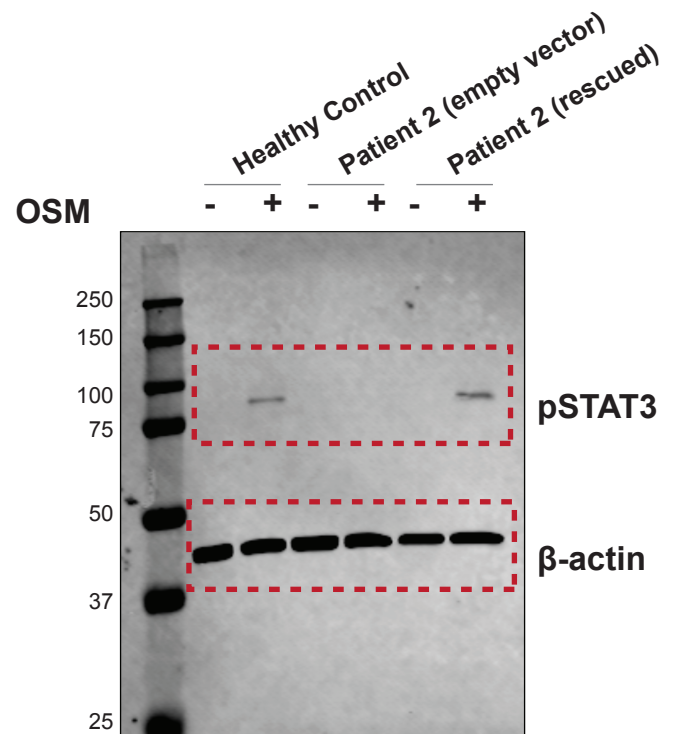

Supplement: SourceData F7 — is the source file for Fig. 7. [file jhi_20260067_sourcedataf7.pdf]

# Source Data for Figure S3

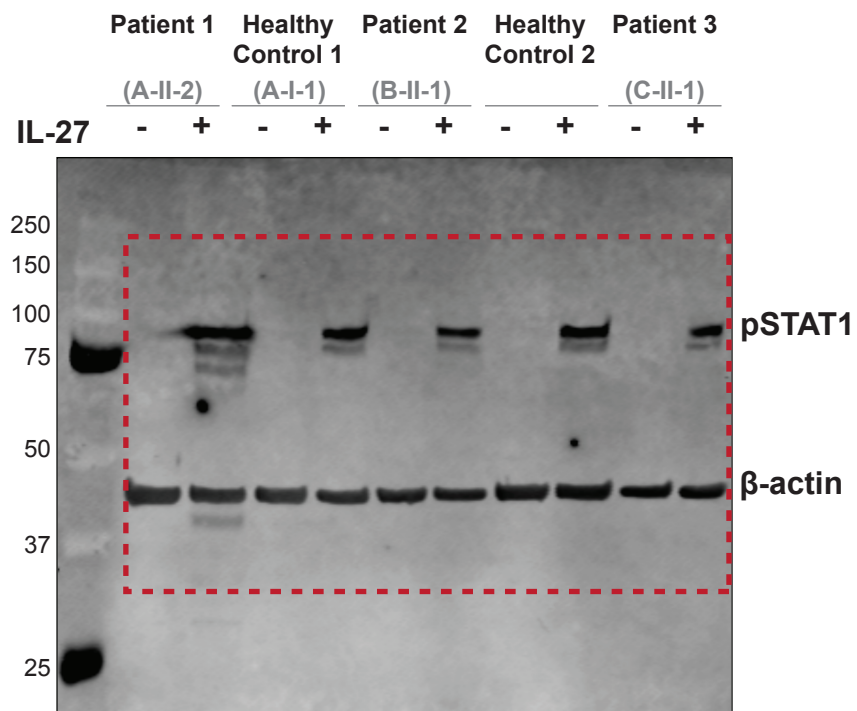

Supplement: SourceData FS3 — is the source file for Fig. S3. [file jhi_20260067_sourcedatafs3.pdf]
